# Assigning Article-Level Themes in Bibliometric Analysis: Mode-Based Mapping Approach Using JMIR Aging Publications

**DOI:** 10.2196/79906

**Published:** 2026-05-28

**Authors:** Sam Yu-Chieh Ho, Kang-Ting Tsai

**Affiliations:** 1 Department of Emergency Medicine, Chi Mei Medical Center, No. 901, Chung Hwa Road, Yung Kung DistTainan, Taiwan; 2 Center for Integrative Medicine, Chi Mei Medical Center, No. 901, Chung Hwa Road, Yung Kung Dist, Tainan, Taiwan; 3 Department of Family Medicine, Chi Mei Medical Center, 901 Chung Hwa Road, Yung Kung Dist, Tainan, 72263, Taiwan, 886 0937399106, 886 937399106; 4 Department of Family Medicine, Well Clinic, No. 302, Yunong Rd, East Dist, Tainan, 701011, Taiwan

**Keywords:** bibliometric analysis, theme assignment, following leader clustering algorithm, FLCA clustering, R programming, visual analytics

## Abstract

**Background:**

Although clustering techniques are commonly used in bibliometric analysis to identify research themes, few studies systematically assign these themes back to individual articles. This gap limits the interpretability of findings and hinders granular, article-level longitudinal analysis.

**Objective:**

This study introduces the Theme Assignment Algorithm for Articles (TAAA), a data-driven framework designed to map clustered themes to individual publications. We demonstrate its utility by identifying dominant research patterns and thematic shifts within JMIR Aging.

**Methods:**

TAAA was applied to 434 JMIR Aging articles published between 2020 and 2025. Keywords were harvested from 3 sources: Web of Science Core Collection (WoSCC) Keywords Plus, author-provided keywords, and abstract-derived terms. These were grouped into thematic clusters using the “following leader clustering algorithm”. The TAAA, implemented via R and a web-based application, determined each article’s primary theme using a statistical model to create a discrete article-level variable. Core themes were identified via h-index computation. Analytical visualization included Kano and Sankey diagrams, alongside volcano plots and heatmaps. The framework’s robustness was further tested by applying a “differentially expressed genes” analogy to map “unknown” core metadata across “known” pre/post publication stages using Cohen kappa as the extent of mapping power.

**Results:**

Analysis across the 3 keyword sources yielded 9, 7, and 9 core themes, respectively. The most prominent themes identified were HEALTH (39.4%), OLDER ADULTS (43.1%), and DEMENTIA (25.3%). Notably, DEMENTIA emerged as a consistent core theme across all sources and visual layers, validating the TAAA’s ability to capture cross-source thematic coherence. The adaptation of dual heatmaps demonstrated the algorithm’s capacity for comparative bibliometric mapping in JMIR Aging. A mapping precision of 0.33 provided quantitative evidence of a 2-stage publication pattern, though the separation between stages was not strictly confined to predefined time intervals.

**Conclusions:**

The TAAA framework provides a replicable, scalable, and interpretable method for article-level thematic assignment. Its ability to uncover consistent research patterns—specifically the dominance of dementia-related studies in JMIR Aging—demonstrates its value for bibliometricians and its potential adaptability to other domains, such as bioinformatics-inspired meta-analyses.

## Introduction

### Overview

JMIR Aging is a peer-reviewed, open-access journal dedicated to advancing digital health, technology, and innovation for older adults [1]. It publishes research on eHealth interventions, gerontechnology, aging populations, and health services aimed at improving care and outcomes for aging societies [2]. Despite this clear mission, the underlying thematic structure of its publications remains underexplored, limiting the ability to systematically trace research foci, emerging trends, and evolving priorities [3]. Understanding the journal’s intellectual landscape requires an approach that integrates bibliometric theory, network clustering, and theme identification into a coherent framework.

### Theoretical Background: Themes and Co-Word Structures in Bibliometrics

In bibliometric research, thematic classification plays a central role in mapping intellectual structures and identifying research frontiers [4-6]. The theoretical foundation lies in co-word analysis, which assumes that the co-occurrence of keywords reflects conceptual proximity among research topics [4-9]. Tools such as Bibliometrix, VOSviewer, and CiteSpace operationalize this principle by constructing keyword networks and identifying clusters that represent thematic communities [7-9].

However, existing methods primarily operate at the cluster level, identifying thematic groupings among keywords rather than assigning a single dominant theme to each article [4-6,10,11]. This limits the interpretability of bibliometric maps and constrains microlevel analyses, such as tracing thematic shifts across journals, institutions, or regions.

### Need for Article-Level Theme Assignment

Although advanced methods such as the following leader clustering algorithm (FLCA) [12] and latent Dirichlet allocation–based topic modeling can uncover latent thematic structures, a key gap remains: Cluster-level theme discovery does not automatically translate into clear, article-level theme representation—for example, mapping text-driven patterns (from item/factor structures) into labels that describe article or person attributes.

To date, few bibliometric models explicitly address this gap with a formal mechanism for assigning cluster-derived themes to individual publications. Without this linkage, thematic labeling becomes less consistent, and cross-sectional or longitudinal analyses are harder to interpret and compare [4,5].

To address this theoretical and methodological gap, an article-level theme assignment algorithm (Theme Assignment Algorithm in Articles [TAAA]) is required. TAAA formalizes the process of identifying each article’s dominant theme from multiple candidate clusters, enhancing both interpretability and reproducibility in bibliometric studies.

### An Application or R-Based Framework for Thematic Assignment and Visualization

Although some prior studies have conceptually mentioned “theme-by-article” analyses [4,5], no standardized nor open-source R-based (or application) implementation currently exists [13]. The proposed framework, TAAA, operationalizes the statistical mode of an article’s keyword cluster assignments to determine its most representative theme (ie, from item/bifactor structures into labels that describe article or person attributes). This method enhances computational efficiency while maintaining theoretical consistency with co-word analysis principles.

Furthermore, the framework integrates visual analytic tools via an application (for the general public) and R-based scripts (for academic researchers), including Kano diagrams [14,15] and Sankey visualizations [11], to illustrate how the newly created theme variable propagates across articles, institutions, and nations. This integration advances existing bibliometric visualization paradigms by enabling article-level interpretability within cluster-based systems.

### Study Aim and Contribution

This study aimed to develop and demonstrate an application and R-based TAAA for assigning dominant themes to publications in JMIR Aging. The contribution of this work is 2-fold:

1. Conceptual: It extends bibliometric theory by linking cluster-level co-word analysis to article-level thematic representation, addressing a critical gap in interpretive granularity.
2. Methodological: It provides a transparent, reproducible, and scalable application or R-based workflow for theme assignment and visualization.

We hypothesized that TAAA can accurately capture each article’s primary thematic focus and improve interpretability in large-scale bibliometric analyses. We further expected the framework to be adaptable to other journals and research domains, as demonstrated through several practical examples.

## Methods

### Data Source

We retrieved metadata for 434 articles published in JMIR Aging from 2020 to 2025 using the Web of Science Core Collection (WoSCC) database. The dataset includes bibliographic information such as author names, affiliations, titles, abstracts, and keywords. Study data are provided in Multimedia Appendix 1. Since all records in WoSCC are publicly available and contain no identifiable patient information, institutional review board approval was not required.

### Definition of Key Terms

To ensure conceptual clarity, the following key terms are defined within the context of this study: keyword source, item testlet, person theme, mapping power (MP), and theme variable.

Keyword source refers to the origin of the keywords used for analysis. This study distinguishes between 3 types: (1) author-provided keywords: terms explicitly selected by the authors; (2) Keywords Plus: terms automatically generated by the WoSCC; and (3) abstract-derived keywords: terms extracted from the article abstracts using a custom R script.

Item testlet draws from educational assessment terminology, where a “testlet” represents a cluster of keywords grouped by a common thematic thread. In this study, testlets were generated using FLCA [12], where each cluster reflects a cohesive research topic within the broader keyword network.

Person theme is the primary topical label assigned to an individual article. Using TAAA [4,5], the “person theme” is determined by identifying the statistical mode (the most frequent keyword cluster) associated with that specific article (or person).

**Figure 1.**
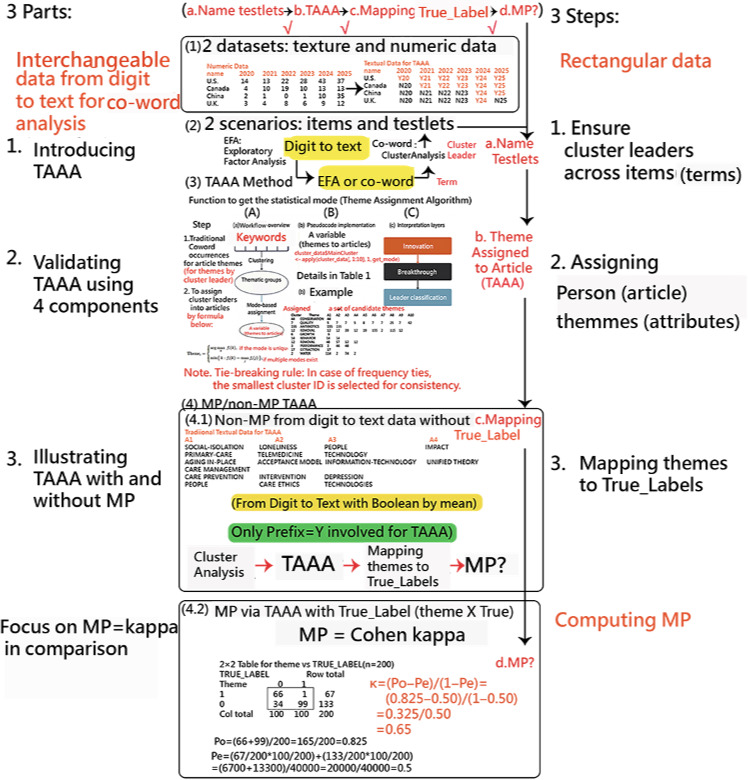
Diagram showing the 3 parts and 3 steps for Theme Assignment Algorithm in Articles (TAAA) in 2 datasets and 2 scenarios (mapping power [MP] vs non-MP with and without True-Label for articles or persons).

MP is a metric used to validate the consistency of thematic classification. As illustrated in Figure 1, MP is computed by mapping keyword clusters (item testlets) to article-level classifications (person themes) and comparing them against established true labels.

For the theme variable, cluster leaders were first generated separately for each keyword source and then merged by mapping item-level terms to person-level themes. This design enables rigorous cross-source comparison and validation of mapping performance against external ground-truth labels (TRUE_LABEL), such as gender or cancer versus noncancer status.

### Summary Performance Report

A clear and concise reporting format focusing on 10 key metadata elements, including top 10 themes, was used to improve readability and interpretation [11], offering an alternative to the extensive graphs and tables commonly found in traditional bibliometric analyses.

### TAAA Applied to This Research

This research consists of 3 parts: (1) introducing TAAA, (2) validating TAAA, and (3) illustrating TAAA with and without MP.

#### Introducing TAAA

##### Creation of a Theme Variable for Co-Word Occurrence Analysis

This study used 3 scenarios to assign themes to articles using 3 keyword sources: Web of Science Keywords Plus, author keywords, and abstract keywords. These were processed via an R script  [16] for the extraction of keywords in articles. Traditionally, article keywords are clustered into thematic groups using algorithms such as FLCA  [12] or tools like CiteSpace  [8], where the largest bubble typically represents the dominant theme in each cluster  [10,11].

The TAAA was further implemented in R  [17], leveraging the statistical mode to identify the most frequently occurring theme among a given article’s clustered keyword assignments based on theme with the top one by count in each cluster. This newly created variable (“theme”) was added to each article for downstream analysis as shown in part 3 in Figure 1 and Textbox 1.

Textbox 1. **Conversion of multiple candidate clusters per article into a single dominant theme with R script.**

# Function: get_mode()

# Purpose : Determine the dominant theme (mode) among candidate clusters

# Context : Used in the Theme Assignment Algorithm for Articles (TAAA) get_mode <- function(x) {

 # Step 1. Clean input data

 # Remove blank entries ("") and missing values (NA)

 x <- x[x != "" & !is.na(x)]

 # Step 2. Handle empty input case

 # If no valid data remain after cleaning, return NA (no theme can be assigned)

 if (length(x) == 0) return(NA)

 # Step 3. Compute frequency table of cluster occurrences

 # The 'table()' function counts how many times each cluster ID appears freq <- table(x)

 # Step 4. Check for tie condition (all clusters occur equally often)

 # If all frequencies are identical, select the smallest cluster number

 # This ensures deterministic (non-random) and reproducible assignment

 if (length(unique(freq)) == 1) {

  return(min(as.numeric(names(freq)))) # smallest cluster ID (tie case)

 # Step 5. Otherwise, return the most frequent cluster (the statistical mode)

 } else {

  return(names(freq)[which.max(freq)]) # dominant cluster (standard mode)

 }

}

#Example usage:

# Apply the function across columns A1–A10 (keyword clusters per article)

# and create a new variable "MainCluster" representing the assigned theme cluster_data$MainCluster <- apply(cluster_data[, 1:10], 1, get_mode)

The approach improves interpretability and computational efficiency by resolving thematic ambiguity, as defined by Equation (1):


(1) $${\text{Theme}}_{i}=\left\{ \begin{array}{ll} \min\left\{k|f_{i}(k)=\max_{j}f_{i}(j)\right\}, & \text{if all }f_{i}(k)\text{ are equal}\\ \text{Mode}({\text{Clusters}}_{\text{keywords}}), & \text{otherwise} \end{array}\right.$$

where $f_{i}(k)$ denotes the frequency of cluster k among the keywords of article i.

For instance, consider an article whose keywords are distributed across 3 clusters—Cluster 1, Cluster 2, and Cluster 3—with respective frequencies of 1, 3, and 2. The algorithm first computes the frequency of each cluster ($f_{i}\left(k\right)$) and identifies Cluster 2 as having the highest count. Therefore, the assigned theme for this article is Cluster 2, as it represents the statistical mode of the clustered keyword assignments.

If all clusters had occurred with equal frequency (eg, 2, 2, 2), the algorithm would select the smallest cluster identifier to ensure deterministic and reproducible assignment of the article’s dominant theme, as shown in part 3 of Figure 1.

For each article, the assigned theme corresponds to the statistical mode of its keyword cluster assignments. In cases of ties (ie, multiple clusters with equal frequency), the cluster with the smallest numerical identifier is selected to ensure consistency and reduce thematic ambiguity.

##### TAAA in Bibliometric Analysis Based on Textual Data

The TAAA framework was applied using visual tools such as Kano  [14,15] and Sankey  [11] diagrams to illustrate thematic distributions across articles, countries, and journals based on co-word occurrence data (eg, “theme” and “country” variables in two columns). The R script  [18] followed methods described in prior studies  [11]. Additionally, leader classification was performed using the dimension coefficient (DC)  [19,20], defining leadership categories as super (DC ≥0.8), quasisuper (DC≥0.7), strong (DC≥0.6), moderate (DC≥0.5), and weak (DC<0.5).

### Venn Diagram Used to Identify the Common Themes From 3 Sources

A Venn diagram was used in R [21] to extract the overlaid themes from the 3 sources: Keywords Plus, author-defined keywords, and abstract. The screened themes denote the core elements in the studied journal.

### Validating TAAA

We evaluated TAAA using 4 complementary validation components: interrater and intrarater validation, external validation and robustness across clustering methods, bootstrapping stability, and out-of-sample holdout robustness.

#### Interrater and Intrarater Validation (Core Theme Subsample)

A stratified subsample was drawn from dominant (“core”) themes (86/431, 20% of core theme articles). Two independent reviewers (2 study authors) who were blinded to TAAA outputs assigned themes using the same predefined label set based on titles and abstracts. Agreement was assessed for TAAA vs Reviewer 1, TAAA vs Reviewer 2, and Reviewer 1 vs Reviewer 2 using confusion matrices and Cohen κ [22], where commonly cited cutoffs [23] are <0.00, poor; 0.00‐0.20, slight; 0.21‐0.40, fair; 0.41‐0.60, moderate; 0.61‐0.80, substantial; and 0.81‐1.00, almost perfect.

#### External Validation and Robustness Across Clustering Methods

Because no universally accepted ground truth exists for topic clustering in keyword co-occurrence networks, we assessed robustness by comparing TAAA assignments under two clustering strategies applied to the same keyword set: (1) FLCA term-to-theme membership from our pipeline (terms_nodes.csv) and (2) Louvain communities from modularity optimization on the keyword co-occurrence graph.

Since cluster counts may differ, we evaluated concordance using (1) a majority-overlap mapping from Louvain communities to FLCA themes based on article-level cross-tabulation and (2) a top-*k* hierarchical criterion in which an article was counted as agreeing if its FLCA theme fell within the top-*k* most frequent FLCA themes inside its Louvain community (*k*=1, 2, 3, 4). Top-1 corresponds to strict one-to-one concordance; larger *k* quantifies hierarchical nesting where a coarse Louvain community may contain multiple FLCA subthemes.

#### Bootstrapping Stability

To assess sampling stability, we performed bootstrap resampling with replacement on the core-theme subsample. For each repetition, κ was recomputed and summarized by its mean and 2.5th-97.5th percentile interval. Jensen-Shannon (JS) distance and mean Spearman correlation across resamples were also computed to quantify stability [24-29].

#### Out-of-Sample Holdout Robustness

To evaluate robustness under dataset shift, we conducted repeated random holdout experiments with a prespecified holdout fraction. On holdout sets, we computed mean Spearman correlation and JS distance between training and holdout rankings to quantify generalization [30,31].

### Illustrating TAAA With and Without MP

#### Textual Terms From Digital Data Assessed Using MP

This text integrates the technical logic of the TAAA with the validation process through MP, supporting both “non-MP” (digit-to-text without true labels) and “MP” (validation against true labels) scenarios to ensure robust bibliometric mapping.

The workflow illustrates the transition in Figure 1, from (1) multisource datasets to (2) the identification of item testlets. (3) The TAAA applies a mode-based function (with tie-breaking rules) to assign a primary person theme to each article (or person). (4) The process concludes with the computation of MP using Cohen kappa [22,23], validating the thematic assignment against true labels to ensure classification accuracy.

#### Steps for Implementing TAAA via R and a Web-Based Application

For the abstract keyword extraction, the input data came from a CSV file named methodsxabstract.csv containing columns for article title and abstract. In R [16] and the application [24], combined text is stored in df$CombinedText. Abstract keywords are extracted and saved to methodsxabstract_with_keywords_topics.csv.

#### TAAA Implementation via R and a Web-Based Application

Keywords across multiple columns are provided in top10keywords.csv. Themes are assigned per article using the TAAA model [17,25] and saved sequentially to methodsxabstract_with_keywords_topics.csv. The resulting “theme” variable is used to analyze co-word occurrences, visualizing thematic distributions across articles, countries, and journals (with variables like “theme” and “country” in two columns).

#### Applications of TAAA for Textual and Numeric Data With MP

The transition from numeric values to article-level (person-level) themes, as shown in part 1 in Figure 1, follows a structured 8-step process:

1. Dichotomization: Convert each numeric item into binary scores using the item-specific mean as a threshold.
2. Tokenization: Transform binary scores into textual tokens by prefixing item labels with “Y” (yes) or “N” (no).
3. Occurrence construction: Retain only “Y-type” tokens to simulate keyword occurrences within each row.
4. Co-word clustering: Apply clustering algorithms to these tokens to derive thematic “cluster leaders” representing coherent testlets across data columns.
5. Person-level assignment: Use the TAAA to map these term-based themes to individual rows (persons), establishing a dominant theme for each entry.
6. External validation (optional): If external reference labels are available, compute MP using Cohen kappa to assess classification accuracy.
7. Web implementation: To enhance user accessibility, the analytical framework [32-34] was deployed as a cloud-based application on Google App Engine (GAE) [35]. This provides a user-friendly interface for researchers to replicate the analysis without requiring local environment configuration, as done with R scripts.
8. Visual interpretation: Analyze the results through an automated HTML report [32,33] featuring Kano diagrams, Sankey plots, volcano plots, and heatmaps.

### Data Sources for Application Examples

The application of TAAA to textual entities via the application was conducted with 434 articles published in JMIR Aging from 2020 to 2025. The application of TAAA to numerical entities via the application occurred via virtual data as a test on STEM and ARTS for men and women and as a questionnaire on 2 domains. In addition, the TAAA was applied to Gene Expression Omnibus (GEO) data via the application: A gene expression profile, GSE8401 [36], including 31 primary melanoma and 52 metastatic melanoma (MM) clinical samples, was downloaded from the GEO database [37]. The differentially expressed genes between MM and primary melanoma were assessed using MP.

### R Packages and Application Used in This Study

All visualizations and data processing were conducted using the R platform  [16,17,20,23] or GAE [35]. Kano diagrams were generated in the application or R scripts, with all statistical analyses performed in R version 4.1.3  [13]. Visualizations and results were produced within the RStudio integrated development environment  [38]. The way to conduct this study using R scripts is described in Multimedia Appendix 2.

## Results

### Summary Performance Report for Top 10 Article Elements

**Figure 2.**
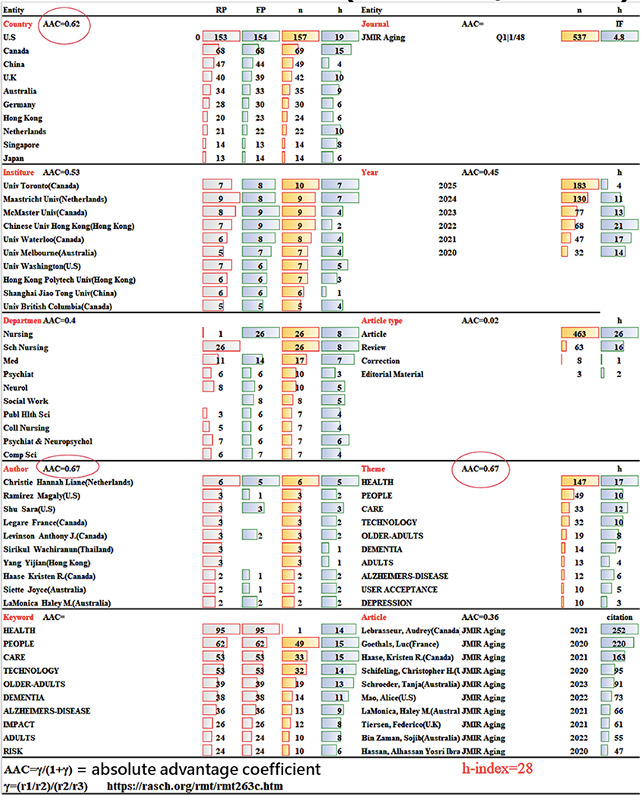
Summary performance report for 10 article metadata categories in JMIR Aging (n=537). FP: first author; RP: first author.

Figure 2 presents a comprehensive summary of 434 publications in JMIR Aging (h-index=28 [39]) analyzed across 10 bibliometric dimensions—country, institution, department, year, article type, author, research area, keyword frequency, and citation count.

The United States, Canada, and the United Kingdom led in publication output, while the University of Toronto (Canada) and Maastricht University (the Netherlands) were among the most productive institutions. Nursing and medical departments contributed prominently, with the majority of papers classified as original research articles.

Notably, Hannah Liane Christie (the Netherlands) was the most prolific author (6 articles [40-45]), whereas Audrey Lebrasseur et al (Canada) produced the most-cited study [46], receiving 252 citations for a rapid review on COVID-19’s impact on older adults. Core keywords such as HEALTH, PEOPLE, CARE, and TECHNOLOGY reflect the journal’s central focus on digital health and aging innovation; see the keyword and theme panels in Figure 2.

Accordingly, this performance overview provides a structural snapshot of JMIR Aging’s research landscape, enabling targeted insight into its geographic reach, institutional strengths, and topical emphasis.

### TAAA for Theme Extraction and Implementation

**Figure 3.**
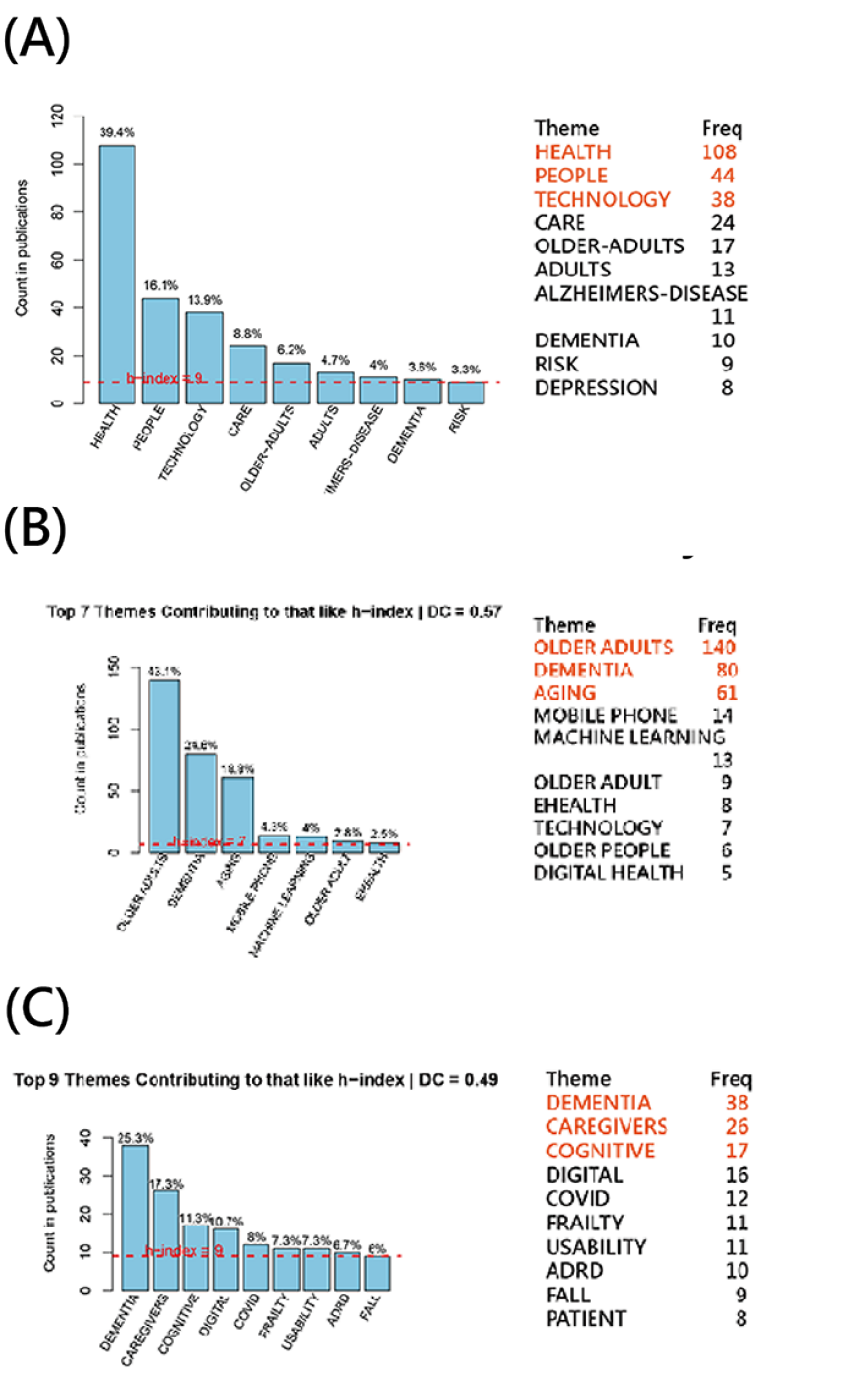
Core themes extracted from JMIR Aging from 3 keyword sources including (A) Keywords Plus, (B) author-defined keywords (top 7 themes contributing to the h-index), and (C) abstract-derived keywords (top 9 themes contributing to the h-index). DC: dimension coefficient.

Figure 3 illustrates how the TAAA identified dominant themes across 3 keyword sources: Keywords Plus (external indexing terms): 9 core themes (Figure 3A); author-supplied keywords: 7 core themes representing self-identified research emphases (Figure 3B); and abstract-derived keywords: 9 themes reflecting content-driven topic extraction (Figure 3C).

Across all 3 scenarios, the top themes were HEALTH (108/282, 39.4%), OLDER ADULTS (140/343, 43.1%), and DEMENTIA (38/158, 25.3%).

**Figure 4.**
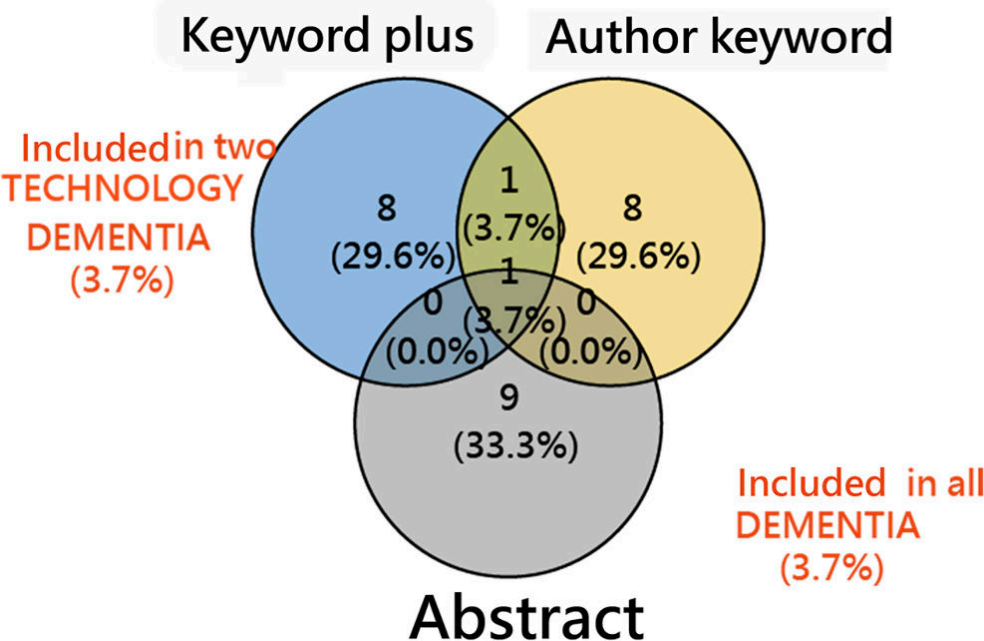
Venn diagram showing DEMENTIA as the only theme overlapping across all 3 TAAA approaches.

To illustrate theme overlap, Figure 4 (Venn diagram) shows that DEMENTIA was the only theme consistently identified in all 3 TAAA runs, underscoring its centrality in the journal’s research portfolio.

Hence, the convergence of DEMENTIA across independent keyword sources validates TAAA’s reliability in identifying dominant thematic foci and highlights the journal’s sustained emphasis on cognitive health in aging research.

### TAAA for Validation

#### Subsample Interrater Validation (Core Themes)

Subsample interrater validation on 57 core theme records (20% sampled from 431 articles) showed substantial agreement between TAAA and Reviewer 1 (κ=0.788; 84.2% exact agreement) and moderate to substantial agreement between TAAA and Reviewer 2 (κ=0.672; 75.4% exact agreement). Human-human agreement was moderate (κ=0.597; 68.4% exact agreement), indicating inherent ambiguity in theme boundary decisions.

Misclassifications were concentrated in a small number of adjacent theme pairs, particularly involving the dominant theme (T1) and conceptually related themes (eg, T13/T15), consistent with expected instability in low-prevalence categories (Table 1).

**Table 1.** Subsample interrater validation for core themes (n=57 core theme records).

	T1^a^	T3^b^	T2^c^	T5^d^	T4^e^	T13^f^	T9^g^	T15^h^	T10^i^
TAAA^j^ versus Reviewer 1 (κ=0.788)									
T1	24	0	0	0	0	1	0	4	0
T3	0	5	0	0	0	0	2	0	0
T2	0	0	7	0	0	0	0	0	0
T5	0	0	0	3	0	0	0	0	0
T4	0	0	0	0	2	0	1	0	0
T13	0	0	0	1	0	1	0	0	0
T9	0	0	0	0	0	0	2	0	0
T15	0	0	0	0	0	0	0	2	0
T10	0	0	0	0	0	0	0	0	2
TAAA versus Reviewer 2 (κ=0.672)									
T1	22	1	0	0	0	2	2	0	2
T3	0	6	0	0	0	0	1	0	0
T2	1	0	6	0	0	0	0	0	0
T5	0	1	0	2	0	0	0	0	0
T4	0	0	0	0	2	0	0	0	1
T13	0	0	0	0	0	1	0	0	1
T9	0	0	0	0	0	1	1	0	0
T15	0	0	0	0	0	0	0	2	0
T10	0	1	0	0	0	0	0	0	1
Reviewer 1 versus Reviewer 2 (κ=0.597)									
T1	18	1	0	0	0	2	2	0	1
T3	0	5	0	0	0	0	0	0	0
T2	1	0	6	0	0	0	0	0	0
T5	0	1	0	2	0	0	0	0	1
T4	0	0	0	0	2	0	0	0	0
T13	1	0	0	0	0	1	0	0	0
T9	0	1	0	0	0	1	2	0	1
T15	3	0	0	0	0	0	0	2	1
T10	0	1	0	0	0	0	0	0	1

a T1: core theme 1.

b T3: core theme 3.

c T2: core theme 2.

d T5: core theme 5.

e T4: core theme 4.

f T13: core theme 13.

g T9: core theme 9.

h T15: core theme 15.

i T10: core theme 10.

j TAAA: Theme Assignment Algorithm in Articles.

When the human-human agreement (κ=0.597) is lower than the TAAA-human agreement (κ=0.788 and 0.672), it leads to several significant implications regarding the reliability and utility of the TAAA algorithm as a “stabilizing force.” Although human reviewers may be influenced by subjective interpretation or fatigue, the TAAA applies a consistent, rule-based mode logic that reduces the “noise” inherent in manual thematic assignment. In bibliometric studies involving hundreds or thousands of articles, manual review is prone to increasing error rates.

#### Agreement Between FLCA and Louvain

Among 431 records, 373 contained at least one keyword. FLCA yielded 53 themes in the term-membership file, whereas Louvain produced 15 communities on the same mapped keyword graph.

Because the two methods differ in granularity, we evaluated hierarchical concordance by allowing each Louvain community to correspond to its Top-*k* most frequent FLCA subthemes. Agreement increased from 49% (183/373; Top-1) to 65.5% (244/373; Top-2), 77.5% (289/373; Top-3), and 82.5% (308/373; Top-4), indicating that most Louvain communities are unions of a small number of FLCA subthemes. To report a chance-corrected measure at the Louvain granularity (*k*=15), we collapsed FLCA themes to Louvain communities via majority overlap at the term level and computed Cohen κ between the resulting labels, yielding κ=0.399, indicating an upper fair effect (0.21‐0.40) [24].

#### Bootstrapping Stability (120 Repetitions)

Across 120 bootstrap resamples, the number of core themes was k=9, and the thematic structure showed high stability (Table 2). The mean JS distance between the bootstrap and reference distributions was 0.057 (SD 0.016), indicating only minor shifts in the theme proportion profile under resampling. The mean Spearman rank correlation was 0.896 (SD 0.071), suggesting that the relative ordering of theme importance was largely preserved across resamples.

**Table 2.** Bootstrapping stability and robustness.

Measurement	Repetitions, n	Core themes, k	Holdout fraction	JS^a^ distance, mean (SD)	Spearman rank, mean (SD)
Bootstrapping	120	9	—^b^	0.06 (0.02)	0.90 (0.07)
OOL^c^ holdout robustness	120	9	0.3	0.12 (0.03)^d^	0.72 (0.13)

a JS: Jensen-Shannon.

b Not applicabe.

c OOL: out-of-likelihood.

d Train, holdout.

#### Out-of-Distribution Holdout Robustness (120 Repetitions)

Under 120 repeated holdout (out-of-likelihood/out-of-sample) evaluations with a 30% holdout fraction, the model retained k=9 core themes (Table 2). The mean JS distance between the training and holdout theme distributions was 0.124 (SD 0.034), which is larger than the bootstrap JS distance, reflecting the expected distributional shift induced by data splitting. The mean Spearman correlation between training and holdout rankings was 0.724 (SD 0.127), indicating moderate agreement in theme ordering and suggesting that the primary thematic structure generalizes reasonably well, while lower-prevalence themes are more sensitive to sample partitioning.

### TAAA for Textual Entities via the Application

**Figure 5.**
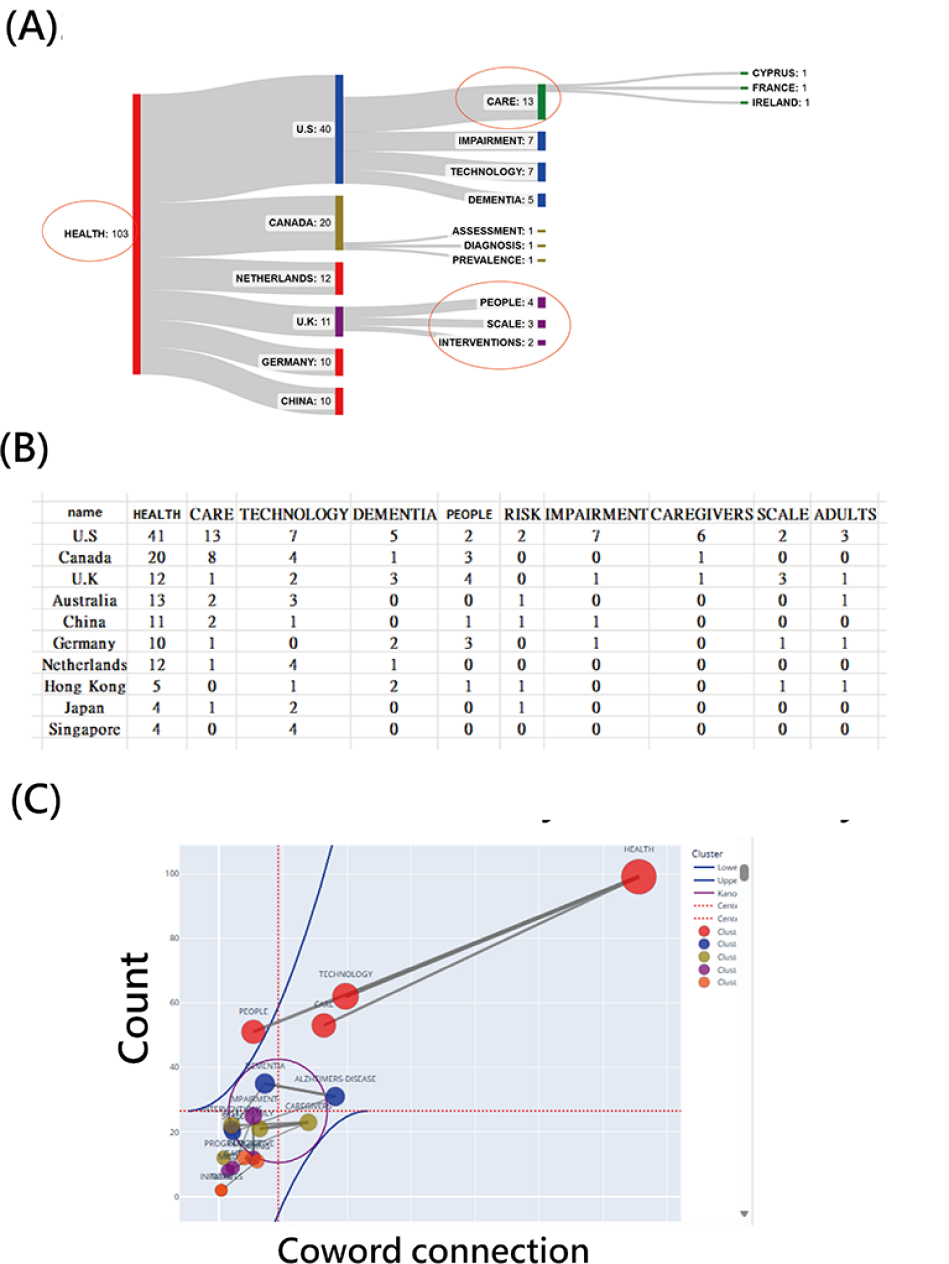
Textual co-word with a new variable of theme: (A) Sankey visualization by country-theme, (B) rectangular data, and (C) create network chart by theme and country via an application [25]. AAC=0.58 <0.7 without dominance role for health=[(99/62)/(62/53)]/(1+[99/62)]/(62/53)]). AAC: absolute advantage coefficient.

Figure 5 demonstrates how assigned themes distribute across countries, highlighting geographic research orientations. The United States remained a research leader, followed by Canada and the United Kingdom.

DEMENTIA and CARE are closely associated with US authors, whereas ASSESSMENT is linked to Canadian research. SCALE and PEOPLE themes are common in UK-based publications. The Sankey diagram in Figure 5A visualizes these relationships—flow width represents the proportional connection between countries and their dominant themes.

In Figure 5B, a network chart of country-theme was uploaded by the input form of rectangular data via an application created by our research team [25,34].

Together, these analyses reveal cross-level variation in thematic prominence—DEMENTIA dominates within specific institutions but shares space with broader aging-related themes at the national level, suggesting specialized research clusters driving global impact.

### TAAA for Numeric Entities via the Application

#### Metadata for Publications in JMIR Aging

Dual heatmaps were constructed to examine metadata structure using complementary selection rules: statistical significance (*P* value) and effect magnitude (log10 fold change). Core metaterms were defined as the union of the top 10 up- and downregulated terms under each criterion. These terms were binarized using the mode of term presence > mean across persons and subjected to co-word analysis, followed by FLCA clustering (target_clusters=2).

Resulting term clusters were propagated to publications via TAAA and displayed beneath each heatmap alongside TRUE_LABEL, reflecting a 2-stage publication structure, with contrast themes denoted by social work versus ALZHEIMERS-DISEASE and editorial material versus China.

**Figure 6.**
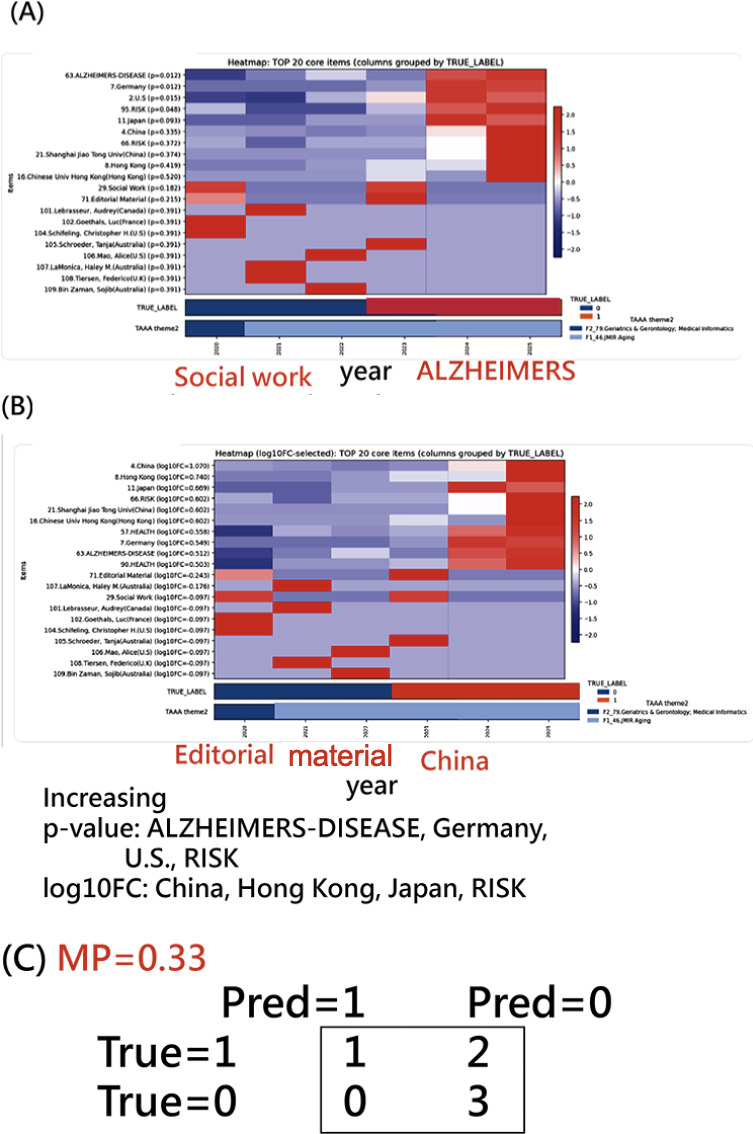
Dual heatmaps for the 66 metaterms in JMIR Aging publications (mapping power [MP]=1.0): (A) heatmap (*P* value–selected) of the top 10 UP + top 10 DOWN metaterms, with increasing *P* values for ALZHEIMERS-DISEASE, Germany, United States, and RISK; (B) heatmap (log10FC-selected) of the top 10 UP + 1op 10 DOWN metaterms, wiith log10FC for China, HongKong, Japan, and RISK; and (C) the probability matrix. TAAA: Theme Assignment Algorithm in Articles.

Although visual correspondence is evident, the modest mapping precision (MP=0.33) indicates residual misalignment between data-driven themes and external labels (Figure 6), suggesting that the thematic transition cannot be strictly partitioned into the periods 2020‐2022 and 2023‐2025.

#### Test on STEM and ARTS for Men and Women via the Application

**Figure 7.**
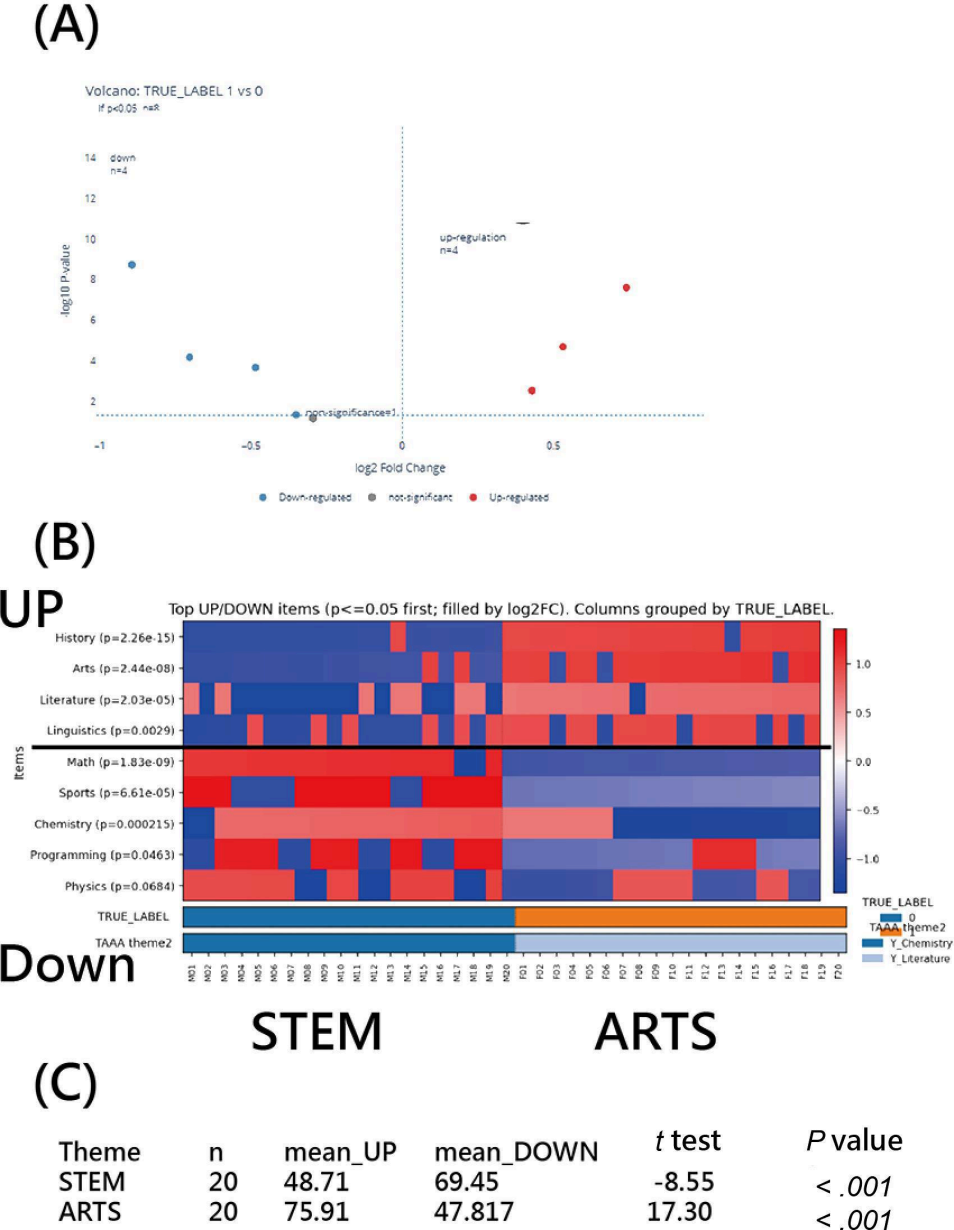
Theme Assignment Algorithm in Articles (TAAA) applied to a test for examinees on STEM and ARTS against gender, shown via a (A) volcano plot, (B) heatmap (κ=1.0), and (C) paired *t* tests within STEM and ARTS.

If the test demonstrated significant discriminatory power in identifying examinees’ attributes [36], as assessed using TAAA against the external reference label (TRUE_LABEL; eg, men vs women), a corresponding volcano plot and heatmap can then be presented, as shown in Figure 7, with perfect agreement between TAAA-derived themes and TRUE_LABEL (MP Cohen κ=1.00).

#### TAAA Applied to a Questionnaire on 2 Domains via the Application

**Figure 8.**
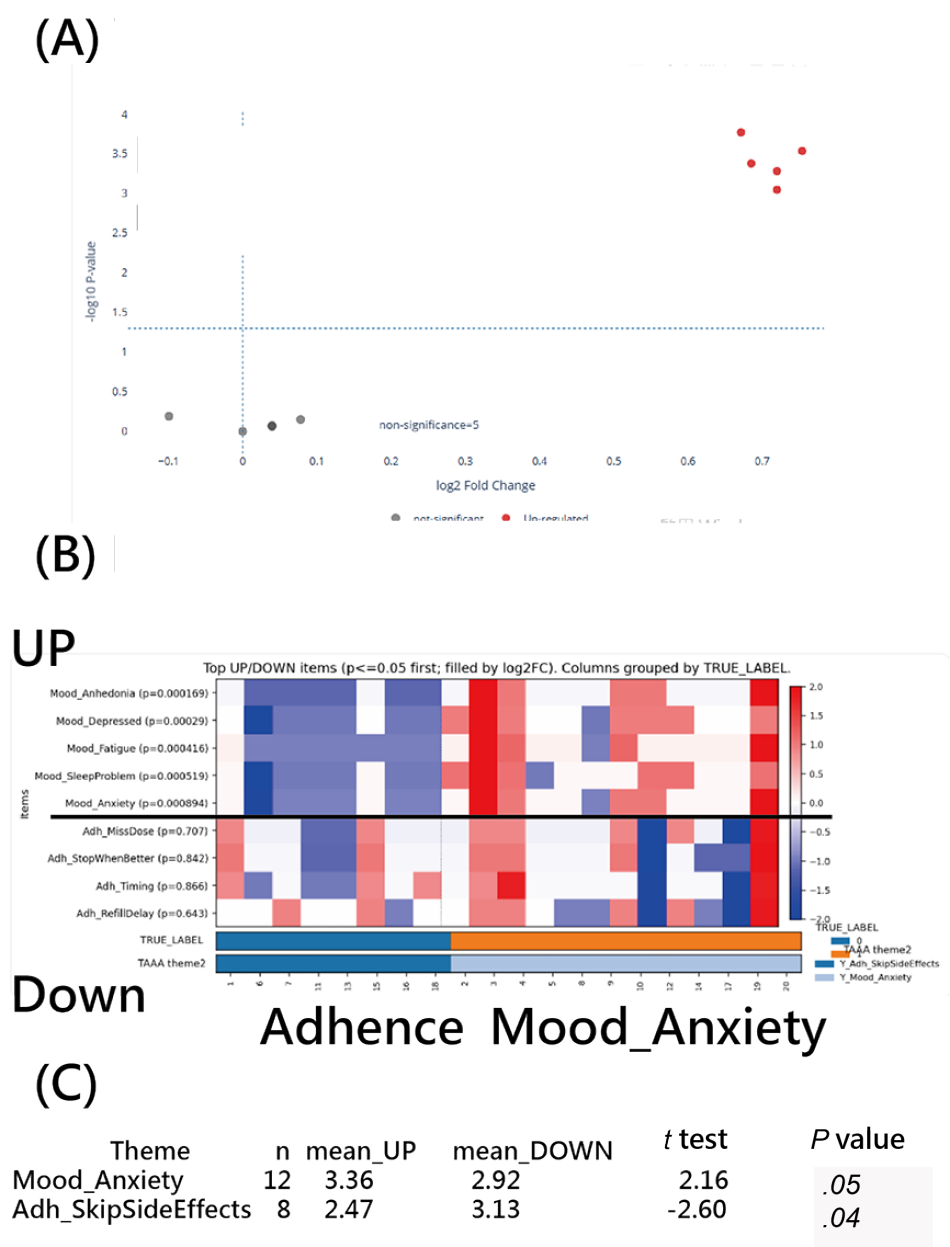
2D scales of the questionnaire on differentially expressed genes of depression/anxiety and medication adherence using Theme Assignment Algorithm for Articles (TAAA): (A) volcano plot, (B) heatmap (κ=1.0), and (C) paired *t* tests within themes.

If the questionnaire demonstrates significant discriminatory power in identifying patient features, as evaluated using TAAA against an external reference label (TRUE_LABEL; eg, depression/anxiety status and medication adherence) [34], the corresponding scree plot and network chart can be presented, as shown in Figure 8, indicating substantial agreement between TAAA-derived themes and TRUE_LABEL (MP: Cohen κ=1.0).

#### TAAA Applied to a Gene Expression Omnibus Data via the Application

**Figure 9.**
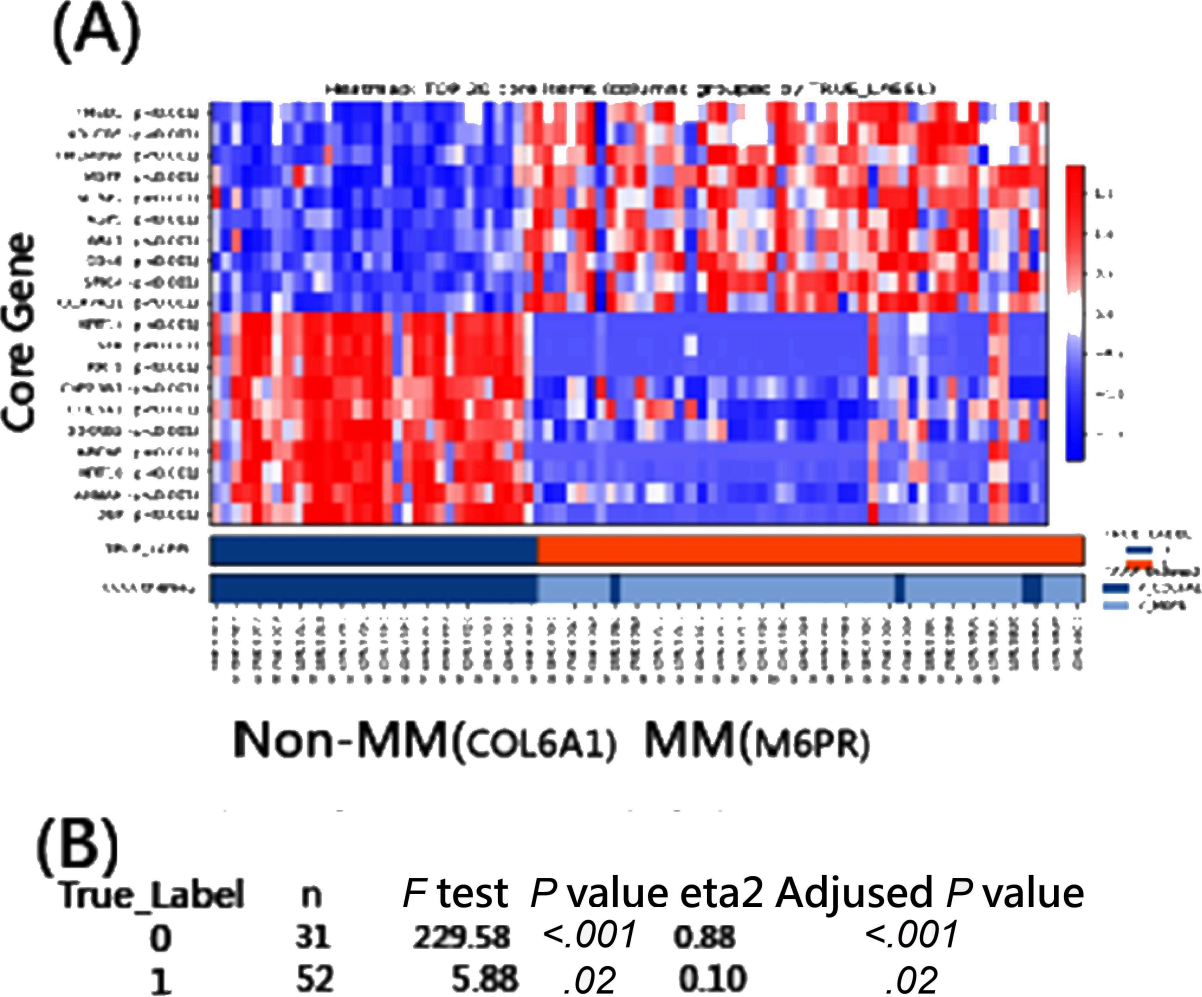
The selection of significant genes for metastatic melanoma (MM) using differentially expressed genes with mapping power (MP)=0.90 based on a smaller *P* value: (A) heatmap (κ=0.90) and (B) paired *t* tests within True_Label.

Figure 9 demonstrates that applying TAAA to the GEO dataset GSE8401 robustly identifies metastasis-related gene patterns distinguishing MM from primary melanoma. The heatmap (MP: κ=0.90) shows a highly consistent separation of samples, with clear up- and downregulation blocks aligned with clinical status, indicating strong agreement between TAAA-derived themes and true labels.

Paired *t* tests within themes further confirmed the biological relevance: The MM and non-MM were statistically significant between gene testlets (*P*<.001 and *P*=.02).

Together, these results validate that, under a stringent MP threshold of 0.90, TAAA effectively selects significant differentially expressed genes and yields interpretable, metastasis-specific gene signatures rather than noisy individual genes—doing what a good method should: turning complexity into clarity with statistical poise.

### Hypothesis Testing Results

The findings of this study confirmed that that the TAAA can reveal meaningful thematic alignments at the journal, country, and institute levels, supporting deeper insights into scholarly trends and research spots, as shown in Table 3.

**Table 3.** Summary of R scripts used in this study with the Theme Assignment Algorithm for Articles (TAAA) model.

Step	Visualization method	R script	Figure	Goal
1	Algorithm for keywords from abstracts	[16,24]	—^a^	Classification
2	Bar plot for core themes and TAAA	[17,25]	Figure 3	Assignment
3	Venn diagram	[22]	Figure 4	Screening
4	Kano and Sankey diagrams	[20,34]	Figures6 7	Leadership
5	Sankey and network	[25]	Figure 5	Network
6	Volcano and heatmap	[34]	Figures6 9	Mapping power

a Not applicable.

Table 4 summarizes key conceptual and methodological differences between TAAA and traditional co-word or cluster-based approaches, underscoring TAAA’s capacity for article-level theme identification and reproducible bibliometric analysis.

**Table 4.** Comparison of Theme Assignment Algorithm for Articles (TAAA) with traditional co-word and theme-assignment methods.

Dimension	Traditional co-word/cluster-based methods	TAAA	Advancement/contribution
Analytical level	Operates at the keyword or cluster level; assigns themes to groups of co-occurring terms	Operates at the article level; assigns a single dominant theme to each article	Enables finer granularity and direct linkage between articles and their thematic focus
Assignment logic	Relies on cluster membership or keyword co-occurrence frequency without article-level aggregation	Uses the statistical mode of clustered keyword assignments within each article to determine its primary theme	Provides a reproducible and deterministic rule for article-level theme labeling
Interpretive output	Generates network maps or clusters without direct theme attribution to individual records	Produces a theme variable for every article, allowing downstream analyses (eg, country, institution, or temporal trends)	Enhances interpretability and enables quantitative comparisons across entities
Reproducibility and transparency	Results vary depending on clustering resolution, software, and visualization parameters.	Fully scripted in R, openly reproducible, with transparent equations and pseudocode (Equation 1)	Improves computational transparency and methodological reproducibility
Scalability and integration	Limited integration with other analytical tools; often visual only	Integrates seamlessly with visualization techniques such as Kano and Sankey diagrams for multidimensional mapping	Supports scalable, automated workflows for journal-, institution-, and country-level analyses

## Discussion

### Principal Findings

The results indicate that the 3 analytical scenarios identified 9, 7, and 9 core themes from their respective keyword sources, with HEALTH (39.4%), OLDER ADULTS (43.1%), and DEMENTIA (25.3%) emerging as the most prevalent themes. Among these, only DEMENTIA was consistently highlighted across all analyses. The application of dual heatmaps demonstrated the algorithm’s capacity for comparative bibliometric mapping in JMIR Aging, with a mapping precision of 0.33 providing quantitative evidence of a 2-stage publication pattern.

These findings support the hypothesis that TAAA effectively assigns a dominant theme to each article using a mode-based approach that aligns with established thematic structures.

- Methodological innovation: Introduces the TAAA, a replicable R-based framework for precise article-level bibliometric mapping
- Multisource validation: Assigns primary themes to 434 JMIR Aging articles using mode-based clustering across 3 distinct keyword sources
- Thematic consistency: Identifies HEALTH, OLDER ADULTS, and DEMENTIA as the dominant research pillars, with dementia showing significant cross-source stability
- Advanced visualization: Uses Kano, Sankey, heatmap, and volcano plots to demonstrate the utility of treating bibliometric metadata with the same rigor as biological “differentially expressed” data

### Adding Knowledge to Literature

This study advances bibliometric methodology by introducing the TAAA [17], which systematically assigns dominant themes at the article level using mode-based clustering, improving on previous techniques  [4,5] with R-based implementation  [16,17]. Unlike traditional keyword co-occurrence analyses that remain at the cluster level (eg, a study  [10] exploring trends and hotspots in research on exercise and Alzheimer’s disease), TAAA enables article-level thematic assignment by adding a dedicated theme column to the dataset. This supports more granular analyses of journal focus, research trends, and associations between article entities.

The application and R-based TAAA framework  [17,32-34] enhances reproducibility and computational efficiency while enabling seamless integration with visualization tools such as Kano and Sankey diagrams  [11,14,15] for clearer thematic mapping with dual heatmaps. Applying TAAA [17,32-34] to JMIR Aging publications demonstrates its practical utility, consistently identifying core themes (eg, DEMENTIA) across multiple keyword sources. Notably, this approach can be applied to other journals to highlight their core themes for readers in future research.

TAAA thus offers a replicable, scalable method for bibliometric studies, supporting country-, institution-, and journal-level analyses that were previously limited by manual theme assignment.

### Editorial Application of Abstract-Derived Keywords

In addition to analytical use, the extraction of keywords directly from article abstracts provides a potential editorial application. By comparing abstract-derived keywords with author-supplied keywords and Keywords Plus terms, editors can evaluate the thematic coherence of submitted or published manuscripts. Discrepancies between the automatically derived and author-assigned keywords may indicate whether an article aligns with the journal’s primary thematic domains or falls outside its intended scope. Such comparison offers a data-driven mechanism for theme validation, assisting editorial boards with maintaining topical consistency, refining journal aims, and identifying emerging research areas within the publication’s portfolio.

### Cross-Level Interpretation of the DEMENTIA Theme

The greater prominence of DEMENTIA observed at the institutional level compared with the country level reflects the localized concentration of specialized research centers and collaborative networks focusing on dementia and cognitive aging. Institutions such as the University of Toronto and Harvard Medical School host long-standing dementia research programs that generate high keyword density and thematic coherence within institutional clusters.

At the national level, however, the thematic landscape becomes more diffuse as research activities span multiple subfields of aging and health technology. This pattern suggests that DEMENTIA functions as a core institutional specialization within certain centers of excellence rather than a uniform national research focus, highlighting TAAA’s capacity to reveal hierarchical differences in thematic intensity across analytical levels.

### Most Cited Article in JMIR Aging

Although Hannah Liane Christie from the Netherlands published the most articles, with 6 publications [24-29] in JMIR Aging, the most highly cited article [46] was authored by Audrey Lebrasseur and colleagues from Canada, with 252 citations. This study with a rapid review synthesizes research on COVID-19’s impact on older adults, revealing increased psychological symptoms, ageism, and reduced social interactions. The findings highlight the need for tailored strategies to maintain social ties and address at-risk older adults’ specific needs.

### Strengths and Implications of This Study

The scalable framework of a reproducible application or R-based TAAA maps co-word clusters to one dominant theme per article using a deterministic mode rule.

Validated mapping via dual heatmaps and MP quantifies alignment with TRUE_LABEL, supporting a stage-like pattern while showing the boundaries are not strictly year-split.

Practical visuals integrating Kano, Sankey/SankeyMATIC, volcano, network, and receiver under the operating curve outputs provide interpretable, actionable bibliometric insights.

### Potential Extensions to Other Domains

Beyond bibliometrics, TAAA can be adapted to clinical and other data-intensive settings where subject-level labels must be inferred from multi-item structures rather than directly observed. In clinical research, bifactor or multidomain measures are often reduced to composite scores and evaluated using logistic regression analysis to predict the patient status. TAAA offers a complementary structure-based alternative: It converts item-level patterns into a discrete subject style by assigning the dominant cluster (mode) across a subject’s activated features, yielding an interpretable 2-style classification suitable for downstream prediction and stratification.

For example, TAAA can be applied to medication records to derive a patient’s dominant treatment pattern (eg, drug class or regimen cluster), supporting analyses of treatment pathways and utilization without requiring explicit brand-company linkage. More broadly, the same framework can harmonize heterogeneous administrative datasets by inferring prevailing categories from fragmented records. These extensions can be implemented using the web-based application [32] provided in this study

### Limitations and Suggestions

First, the analysis relied primarily on Keywords Plus from the WoSCC, which may omit domain-specific concepts or vary in granularity compared with controlled vocabularies such as MeSH used in PubMed. Future studies could integrate multiple bibliographic databases and standardized ontologies to improve thematic coverage and semantic precision.

Second, the mode-based theme assignment in TAAA simplifies articles to a single dominant theme, which may underrepresent genuinely multitopic publications. Extensions incorporating weighted frequencies or probabilistic theme assignments could better capture thematic complexity and are available for further development, as outlined in our GitHub repository.

Third, although abstract- and title-based keyword extraction was automated using R [16] and the application [32], certain preprocessing steps (eg, keyword normalization and cluster interpretation) may still require expert oversight. Greater automation of preprocessing and quality control would further enhance scalability and reproducibility.

Fourth, interrater validation uses two study authors, acknowledges potential expectation bias, and describes blinding procedures more explicitly. Future studies could consider adding more experts to assign themes to articles in comparison withd TAAA.

Finally, validation was conducted using a single journal (JMIR Aging), limiting generalizability. Future work will apply TAAA across multiple journals and domains to more fully assess its robustness, scalability, and cross-disciplinary applicability.

### Conclusions

This study introduces the application or R-based TAAA as a systematic approach for assigning dominant themes at the article level. Application to JMIR Aging publications demonstrates that TAAA enhances interpretability and reproducibility while enabling nuanced bibliometric analyses across journals, countries, and institutions. By bridging cluster-level co-word structures with article-level thematic representation, TAAA provides a transparent framework for large-scale thematic mapping. Future work may extend this approach by integrating semantic embeddings or applying the framework to multidisciplinary datasets.

## Supplementary material

10.2196/79906 Multimedia Appendix 1 Dataset used in this study.

10.2196/79906 Multimedia Appendix 2 How to conduct this study.

## Abbreviations

DC: dimension coefficient
FLCA: following leader clustering algorithm
GAE: Google App Engine
GEO: Gene Expression Omnibus
JS: Jensen-Shannon
MM: metastatic melanoma
MP: mapping power
TAAA: Theme Assignment Algorithm in Articles
WoSCC: Web of Science Core Collection
