# Supplementary material for: Assigning Article-Level Themes in Bibliometric Analysis: Mode-Based Mapping Approach Using JMIR Aging Publications
Source: JMIR Aging. 2026 May 28;9:e79906. doi: 10.2196/79906 (PMC13218566; doi:10.2196/79906)
Supplement: Multimedia Appendix 2 [file aging-v9-e79906-s002.pdf]

https://taaakappa-dot-taaacoword2512.df.r.appspot.com/

🌐 TAAA Semantic–Co-Word Analyzer (1st and last columns are name and True labels

Multilingual Semantic Network & Theme Assignment

🚀 0. 10 Steps in Excel for WoS

📥 **Download Sample Datasets**

[illegible]

TAAA from Digital → Text with mapping power(MP=kappa) vs True_Label

Run

選擇檔案 geneskin.csv

Dummy patient values (comma-separated; same order as item columns). Leave empty → use top-1 row.

80,70,60,20,20,30,72,55,40

Detected item column order (from uploaded CSV/TSV/TXT header):

Gene GSM207929 GSM207930 GSM207931 GSM207932 GSM207933 GSM207934 GSM207935 GSM207936 GSM207937 GSM207938 GSM207939 GSM207940 GSM207941 GSM207942 GSM207943 GSM207944 GSM207945 GSM207946 GSM207947 GSM207948 GSM207949 GSM207950 GSM207951 GSM207952 GSM207953 GSM207954 GSM207955 GSM207956 GSM207957 ... (+54)

Enter values in this order (84 items). Blank means skip.

Run (Upload) Run Demo Status: Success! Loading report...

Report: /reports/cfbd7e943669/report.html

Report preview

Heatmap shows 20 items × 83 persons (x truncated if too wide).

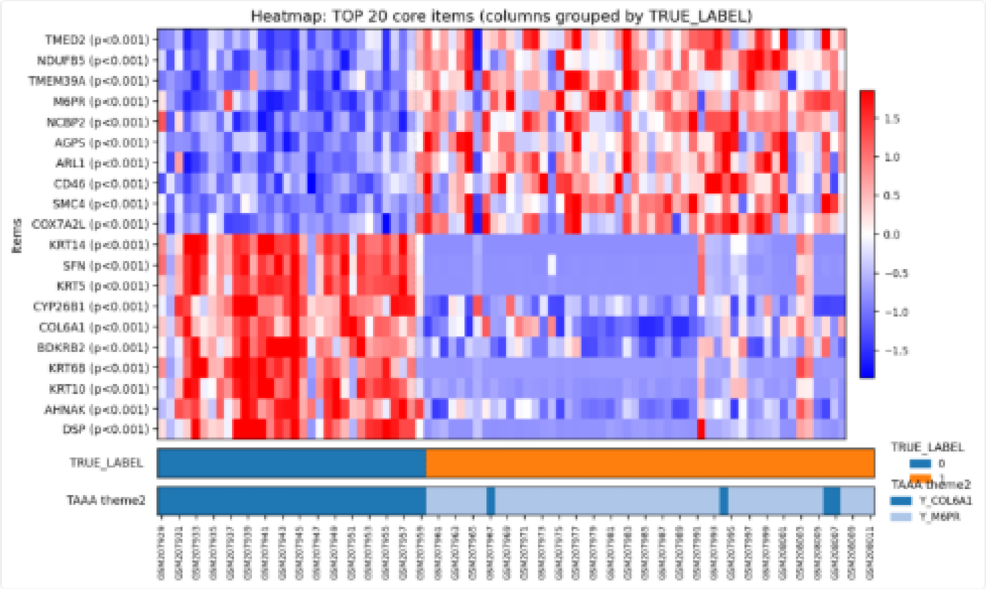

Kappa(TAAA theme2 vs TRUE_LABEL): 0.900 (agreement: 0.952, n_used: 83, excluded_unassigned: 0) (mapping: Y_COL6A1→0, Y_M6PR→1)

2×2 table (TRUE_LABEL vs mapped theme2)

	Pred=1
True=1	48
True=0	0

Classification metrics

Accuracy	
Precision	
Recall (Sensitivity)	
Specificity	
F1	
AUC (hard)	

Contingency: TRUE_LABEL × theme2 (excluding Unassigned)

Volcano plot (interactive)

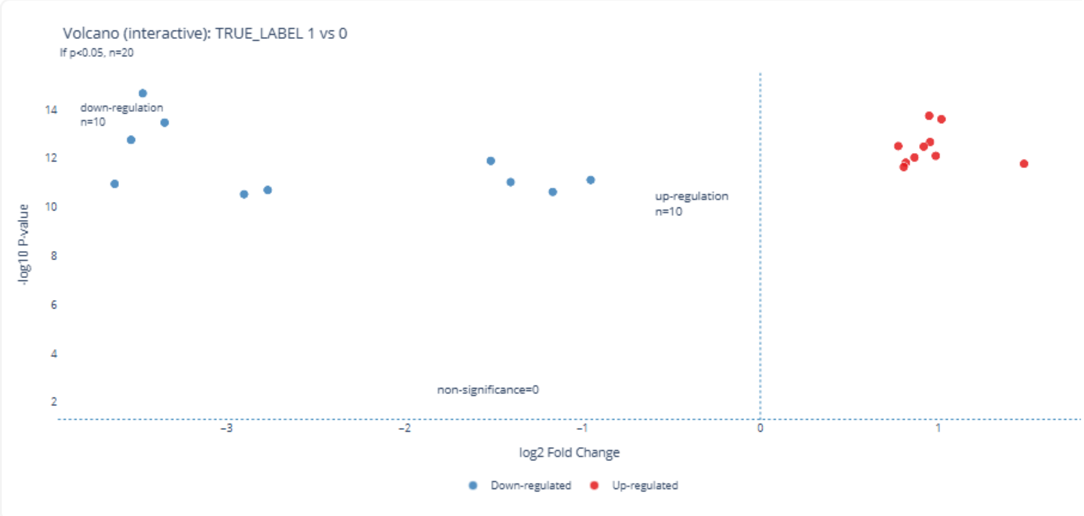

TAAA

Upload field name is `file`. Demo uses `/demo` (no upload needed). Report is shown below (no pop-up).

選擇檔案 jmirJAage.csv | Upload & Run (No C/D) | Upload & Run (With C/D) | Demo (No C/D) | Demo (With C/D)

⏱ co-word: ~5–8 min

https://taaa-dot-taaacoword2512.df.r.appspot.com/

After 7 minutes and 17 seconds, html results are provided along with bootstrapping and OOL robustness and stability validation.

Report — open in new tab run_id: d4346b3

Columns used for extraction	A1 A2 A3 A4 A5 A6 A7 A8 A9 A10 A11 A12 A13 Unnamed: 13 Unnamed: 14 Unnamed: 15 Unnamed: 16 Unnamed: 17 Unnamed: 18
Articles (total rows)	431
Articles with ≥1 extracted keyword	373
Assigned (has matched terms)	373
Unassigned (cluster = -1)	58
...of which: no extracted keywords	58
...of which: has keywords	0

先用 Keywords Plus 建共現網路 → Louvain 分群（共 14 群）

每篇文章先從 Keywords Plus 推得「可能 themes」

- **若唯一** → 直接選
- **若多個** → 先看該篇所屬 Louvain 群的 **cluster mode theme（同群眾數/眾數）** 做優先（mod），再用 **Title+Abstract** 做語意決選

仍遵守：**最後選到的 theme 必須落在 Keywords Plus 推得的 themes**；不夠貼合則標 weak_fit

Prompt to ChatGPT for theme in selection from 9 core themes

You are a careful semantic classifier. Assign EXACTLY ONE theme from the 9 labels:

HEALTH, CARE, TECHNOLOGY, DEMENTIA, PEOPLE, RISK, IMPAIRMENT, CAREGIVERS, SCALE.

THEME DEFINITIONS (use these scopes; pick best semantic fit)

1) HEALTH: General health status, prevention, wellbeing, comorbidities, symptoms, outcomes (not dementia-specific unless clearly central).

2) CARE: Healthcare delivery/practice, interventions, services, care pathways, quality of care, care settings, policy/implementation.

3) TECHNOLOGY: Digital health, AI/ML, sensors, apps, telehealth, assistive tech, data systems used to assess/support care/health.

4) DEMENTIA: Dementia/Alzheimer's/cognitive disorders as the central condition (diagnosis, progression, management, epidemiology).

5) PEOPLE: Population characteristics, demographics, experiences, attitudes, behaviors, engagement, qualitative lived experience (not primarily caregivers).

6) RISK: Risk factors, prediction, screening/risk stratification, odds/associations, incidence determinants.

7) IMPAIRMENT: Functional/cognitive/physical impairment, disability, ADL/IADL limitations, decline measures (when impairment is the focus, not just a symptom).

8) CAREGIVERS: Informal/formal caregivers, caregiver burden/stress, support needs, caregiver interventions, dyads.

9) SCALE: Scale development/validation, psychometrics, reliability/validity, questionnaire/instrument creation, scoring, measurement properties.

You are a careful semantic classifier. Assign EXACTLY ONE theme from the 9 labels:

HEALTH, CARE, TECHNOLOGY, DEMENTIA, PEOPLE, RISK, IMPAIRMENT, CAREGIVERS, SCALE.

TASK and DECISION RULES via TAAA

1. Referred to the cluster results(do not uses the *real Louvain result)*:

**theme**	**leader**	**top_terms_with_freq**	**n_terms**
1	HEALTH	HEALTH(99); BURDEN(19); INTERVENTION(16); QUALITY-OF-LIFE(16); BARRIERS(14);	235

theme	leader	top_terms_with_freq	n_terms
		LIFE(14); AGE(12); BEHAVIOR(12); OUTCOMES(12); QUALITY(12); SYMPTOMS(12); TELEMEDICINE(12); INTERNET(9); PREVENTION(9); ADOPTION(8); EFFICACY(8); FALLS(8); FRAMEWORK(8); IMPLEMENTATION(8); MOBILITY(8); STRATEGIES(8); UNITED-STATES(8); ACTIVITIES(7);	

theme	leader	top_terms_with_freq	n_terms
		CARERS(7); DISABILITY(7); KNOWLEDGE(7); INSTRUMENTAL(6); MASS(6); METAANALYSIS(6); PREDICTORS(6)	
2	TECHNOLOGY	TECHNOLOGY(62); TELEHEALTH(11); PERCEPTIONS(10); ASSISTIVE(7); BENEFITS(5); COVID-19(5); EDUCATION(4); INFORMAL(4); LANGUAGE(4); PERSPECTIVES(4);	56

theme	leader	top_terms_with_freq	n_terms
		CONTROL(3); EVERYDAY(3); MENTAL-STATE-EXAMINATION(3); QUESTIONNAIRE(3); TELECARE(3); VIRTUAL-REALITY(3); CLINICAL-TRIAL(2); CONNECTEDNESS(2); DISORDER(2); EASE(2); END(2); IDENTIFICATION(2); MEDICINE(2); MODERATE(2); MOTIVATION(2); OF-LIFE(2); PRIMARY-	

theme	leader	top_terms_with_freq	n_terms
		CARE(2); PRIVACY(2); SCALES(2); SURVEILLANCE(2)	
3	CARE	CARE(53); RESIDENTS(13); TECHNOLOGIES(10); DESIGN(8); SELF-MANAGEMENT(8); SYSTEM(7); NURSING-HOME(6); SMART(5); THERAPY(5); HEART-FAILURE(4); HOMES(4); TRIAL(4); CANCER(3); ETHICS(3); CENTERED(2); DECISION(2);	67

theme	leader	top_terms_with_freq	n_terms
		DISCHARGE(2); ENGAGEMENT(2); HOSPITALIZATIONS(2); INDEPENDENCE(2); OBSTRUCTIVE(2); PULMONARY-DISEASE(2); RESIDENTIAL(2); SYSTEMS(2); 3-MINUTE(1); 3D-CAM(1); ADAPTATIONS(1); AFTER-DISCHARGE(1); AGEISM(1); AGITATION(1)	
4	PEOPLE	PEOPLE(51); COMMUNITY(13);	44

theme	leader	top_terms_with_freq	n_terms
		EXPERIENCES(8); BALANCE(5); LONG-TERM-CARE(4); PSYCHOLOGICAL(3); CAPACITY(2); DEATH(2); RETENTION(2); SERVICE(2); TOOLS(2); AFRICAN-AMERICANS(1); AGILITY(1); AUGMENTED(1); CAMBERWELL(1); CANE(1); CHAIR-STAND(1); CLAIMS(1); CLINICAL-	

theme	leader	top_terms_with_freq	n_terms
		OUTCOMES(1); CO-DESIGN(1); DECISIONAL(1); DEMNET-D(1); DISORIENTATION(1); DYNAMIC(1); GAMES(1); GENERAL-POPULATION(1); HELPLINE(1); IMPROVES(1); INCIDENTS(1); INSTITUTIONALIZATION(1)	
5	DEMENTIA	DEMENTIA(35); DECLINE(9); MEMORY(3); BRAIN(2);	32

theme	leader	top_terms_with_freq	n_terms
		CHOLESTEROL(2); DRIVERS(2); ELDERS(2); LOST(2); MINI-MENTAL-STATE(2); ACTIVATED(1); ASSOCIATIONS(1); BONE(1); COMPLEXITY(1); CONCURRENT(1); CORTICAL(1); CROSS-VALIDATION(1); DIABETES-MELLITUS(1); EAR(1); FUNCTIONS(1); GETTING(1); HEALTH-	

theme	leader	top_terms_with_freq	n_terms
		PROMOTION(1); IMPLICIT(1); KINECT(1); MULTICOMPONENT(1); MULTIDOMAIN(1); ONE(1); RECORDER(1); VETERANS(1); WANDERING(1); WEB-BASED(1)	
6	OLDER-ADULTS	OLDER-ADULTS(35); SOCIAL(6); ROBOTS(4); AGED(3); ELDERLY(3); SCORE(2); VISUAL(2); ABILITIES(1); ATTENTION(1); CARDIAC-SURGERY(1);	21

theme	leader	top_terms_with_freq	n_terms
		CONFIDENCE(1); CONSENSUS(1); EXPOSURE(1); FEEDBACK(1); FRACTURES(1); PARO(1); SOCIETY(1); STATES(1); STENOSIS(1); SUSTAINABILITY(1); TAVI(1)	
7	ALZHEIMERS-DISEASE	ALZHEIMERS-DISEASE(31); BEHAVIORAL(3); CORNELL(2); DIAGNOSTIC-CRITERIA(2); MOCA(2);	10

theme	leader	top_terms_with_freq	n_terms
		SPEECH(2); CONNECTED(1); PROGRESSION(1); PROVISIONAL(1); PSYCHOPATHOLOGY(1)	
8	IMPACT	IMPACT(26); CLINICAL-TRIALS(3); DEVICES(3); FATIGUE(2); HIV(2); ALTERNATIVE(1); BOTTLE(1); CESSATION(1); DRIVING(1); HEIQ(1); LEADERSHIP(1); PILL(1); RECALL(1); SCOPE(1); TAI-CHI(1)	15

theme	leader	top_terms_with_freq	n_terms
9	IMPAIRMENT	IMPAIRMENT(25); COGNITIVE(12); MILD(8); FUNCTION(3); WORKING-MEMORY(3); TRAIL(2); ACTION(1); AFRICAN-AMERICAN(1); ALZHEIMER-DISEASE(1); DISCOVERY(1); INTRAINDIVIDUAL(1); KATZ(1); LOWER-EXTREMITY(1); TABLES(1); TASK(1); TERM(1); VISION(1); VISUAL-ATTENTION(1)	18

theme	leader	top_terms_with_freq	n_terms
10	CAREGIVERS	CAREGIVERS(23); FAMILY(21); PROGRAM(12); STRESS(12); CONTROLLED-TRIAL(6); PHYSICAL(4); RANDOMIZED(4); PROCESS(2); MEDITATION(1); REDUCTION(1)	10
11	INTERVENTIONS	INTERVENTIONS(22); POLYPHARMACY(2); UPDATE(1)	3
12	ACCEPTANCE	ACCEPTANCE(21); USER(11);	9

theme	leader	top_terms_with_freq	n_terms
		PERSPECTIVE(4); DETERMINANTS(2); LIFE-COURSE(2); MHEALTH(2); EXPLORATORY(1); FACTOR-ANALYSIS(1); SAMPLE-SIZE(1)	
13	RISK	RISK(21); INDEX(5); SIZE(3); BIOMARKERS(2); FITNESS(2); HOSPITALIZATION(2); PRESSURE(2); SELECTION(2); BODY-MASS(1); CARDIORESPIRATORY(	28

theme	leader	top_terms_with_freq	n_terms
		1); CARDIOVASCULAR(1); CIGARETTE-SMOKING(1); CIRCUMFERENCE(1); COHORT(1); DOCUMENTATION(1); ENVIRONMENT(1); FACILITATE(1); GENES(1); IDENTIFYING(1); OS(1); OXIDATIVE(1); PERIMENOPAUSAL(1); PHYSICAL-FITNESS(1); PREDICTOR(1); SURGERY(1);	

theme	leader	top_terms_with_freq	n_terms
		SURVIVAL(1); ULCER(1); WAIST(1)	
14	ADULTS	ADULTS(20); OLDER-PEOPLE(5); SEVERITY(2); ARTHRITIS(1); ASIAN-AMERICANS(1); ETHNIC-DIFFERENCES(1); FASTING(1); GLUCOSE(1); GLYCEMIC(1); HYPERTENSION(1); IMPAIRED(1); MULTIFACTORIAL(1); MUSCLE(1); OLDER(1);	19

theme	leader	top_terms_with_freq	n_terms
		POSITION(1); PREDICT(1); QUALITY-OF-CARE(1); SIGNS(1); WEIGHT-LOSS(1)	
15	SCALE	SCALE(20); NEUROPSYCHIATRIC(6); INVENTORY(5); MENTAL-HEALTH(5); NETWORK(3); PROPERTIES(2); PSYCHOMETRIC(2); ACHIEVEMENT(1); AVOIDANCE(1); BRADEN(1); CENTER(1); CULTURAL-	21

theme	leader	top_terms_with_freq	n_terms
		ADAPTATION(1); DISTRESS(1); ECOLOGICAL(1); INFORMATION-SEEKING(1); MOMENTARY(1); MORALE(1); MULTISITE(1); OVERLAP(1); SEARCHES(1); SHORT-FORM(1)	
16	SUPPORT	SUPPORT(20); IMPROVEMENT(2); INNOVATIONS(2); TELEPHONE(2); CONTENT(1);	7

theme	leader	top_terms_with_freq	n_terms
		DISTANCE(1); MULTIMEDIA(1)	
17	DEPRESSION	DEPRESSION(19); ANXIETY(8); AIDS(1); BCAT(1); COGNITIVE-BEHAVIORAL(1); INFECTION(1); SEX-DIFFERENCES(1)	7
18	VALIDATION	VALIDATION(19); VERSION(7); CHINESE(5); HONG-KONG(2); INITIAL(2); SAMPLE(2); CODES(1); DEFINITIONS(1); LIFE-SPAN(1); MEDICAID(1); SCREENING(1);	14

theme	leader	top_terms_with_freq	n_terms
		SEGREGATION(1); TEST-RETEST(1); US(1)	
19	ASSOCIATION	ASSOCIATION(18); ALZHEIMERS(7); DIAGNOSTIC(4); INSTITUTE(3); NATIONAL(3); RECOMMENDATIONS(3); SARCOPENIA(3); WORKGROUPS(3); APOLIPOPROTEIN-E(1); GENOTYPE(1); PHENOTYPE(1); SEX(1)	12
20	PREVALENCE	PREVALENCE(18); TIME(4); SEGMENTATION(2);	5

theme	leader	top_terms_with_freq	n_terms
		NUTRITIONAL(1); PHOTOGRAPHY(1)	

2. In uploaded file, if keyword plus has space between words, then replace the space with hythen between them>

3. Comparing keywords plus to the 9 themes each, if unique, select the unique theme, otherwise, referred to other keywords plus with mod(possible themes in identical cluster), ie, **Within-row mode** using only that article’s keywords that belong to that cluster, if tied, select the smaller number of cluster, and select the single most suitable theme from those 9 themes mentioned above as cluster leaders, respectively, based on overall cluster analysis.

2. The selected theme must be one of keyword plus in the 2nd column.

3. If multiple themes apply, choose the DOMINANT one. Tie-breaker:

(a) choose the most specific scope; (b) if still tied, choose the earlier item in the list above.

4. If none fits well, choose the closest theme from one of themes in 2nd column, but mark weak_fit with low confidence.

5. if the classification is different from TAAA in the 3rd column, give the reason for it.

- Do not add facts not present in the text. fill the selected theme into 1st column based on title, abstract, and keywords plus in 2nd to 4th columns in the provided csv file , with a downloadable cvs file for me.

TAA in Chinese

用 theme_assignment (37).csv 的 top_terms_with_freq 建立 **keyword → theme(群號)** 對照

每一列（row）把 theme_article (6).csv 的 keywords（第 2 欄到最後一欄）對照到 theme

用 **mod(theme)**（出現次數最多的 theme）當該列的 final theme

若 tie：選較小的 theme number

把算出的 **theme leader（對應該 theme 的 leader）** 填進 theme_article (6).csv 的第 **1 欄**

結果：不是 100%，是 99.73%

1 列不一致（而且剛好是 **tie case**：兩個 theme 票數一樣，你指定的 tie-break「選較小 theme number」會選到另一個 leader）

TAAA in English:

To ensure reproducibility and to preserve phrase integrity, each keyword cell was treated as a single phrase (no further splitting by whitespace or punctuation). For each

article, we computed its theme as the **mode** of the cluster IDs of its constituent keywords. When ties occurred, the article was assigned to the **smaller** cluster ID. Only records containing at least one mappable keyword (i.e., a keyword appearing in the FLCA term list) were included in the comparison.

Top-k hierarchical concordance

Because FLCA and Louvain may yield different numbers of clusters and different granularity, strict one-to-one label matching can be overly conservative or even misleading. We therefore evaluated cross-method robustness using a **Top-k hierarchical concordance** criterion that allows a coarse community to correspond to multiple fine-grained sub-themes.

Let $i = 1, \ldots, n$ index articles. Let $L_i \in \{1, \ldots, K_L\}$ denote the Louvain community of article i, and let $F_i \in \{1, \ldots, K_F\}$ denote its FLCA theme. F_i is obtained via the TAAA rule: $F_i = \arg\max_t c_{it}$, where c_{it} is the count of keywords of article i belonging to FLCA theme t; ties are resolved by choosing the smaller theme ID.

For each Louvain community l, define the empirical overlap counts:

$$N_{l,t} = \sum_{i=1}^{n} \mathbf{1}(L_i = l)\mathbf{1}(F_i = t),$$

and let $\mathcal{T}_k(l)$ be the set of the k FLCA themes with the largest $N_{l,t}$ (ties broken deterministically).

We define the **Top-k agreement** as:

$$A_k = \frac{1}{n}\sum_{i=1}^{n} \mathbf{1}(F_i \in \mathcal{T}_k(L_i)), k \in \{1,2,3,4\}.$$

Here, A_1 corresponds to strict one-to-one concordance (each Louvain community mapped to a single most-overlapping FLCA theme), while A_k with $k > 1$ quantifies hierarchical nesting (a Louvain community representing a union of up to k FLCA sub-themes).

To avoid overstating agreement, we emphasize A_1 and A_2 as primary robustness measures and report A_3–A_4 as sensitivity analyses.

Chance-corrected and label-invariant indices

In addition to Top-k agreement, we report complementary indices that address chance agreement and label permutations. After mapping each Louvain community l to its single most-overlapping FLCA theme $m(l) = \arg\max_t N_{l,t}$, we compute **Cohen's** κ between F_i and $m(L_i)$ as a chance-corrected agreement measure. We also report **Adjusted Rand Index (ARI)** and **Normalized Mutual Information (NMI)** between the un-mapped labelings $\{F_i\}$ and $\{L_i\}$, as these metrics are invariant to label permutations and do not require equal numbers of clusters.

Top 20 terms

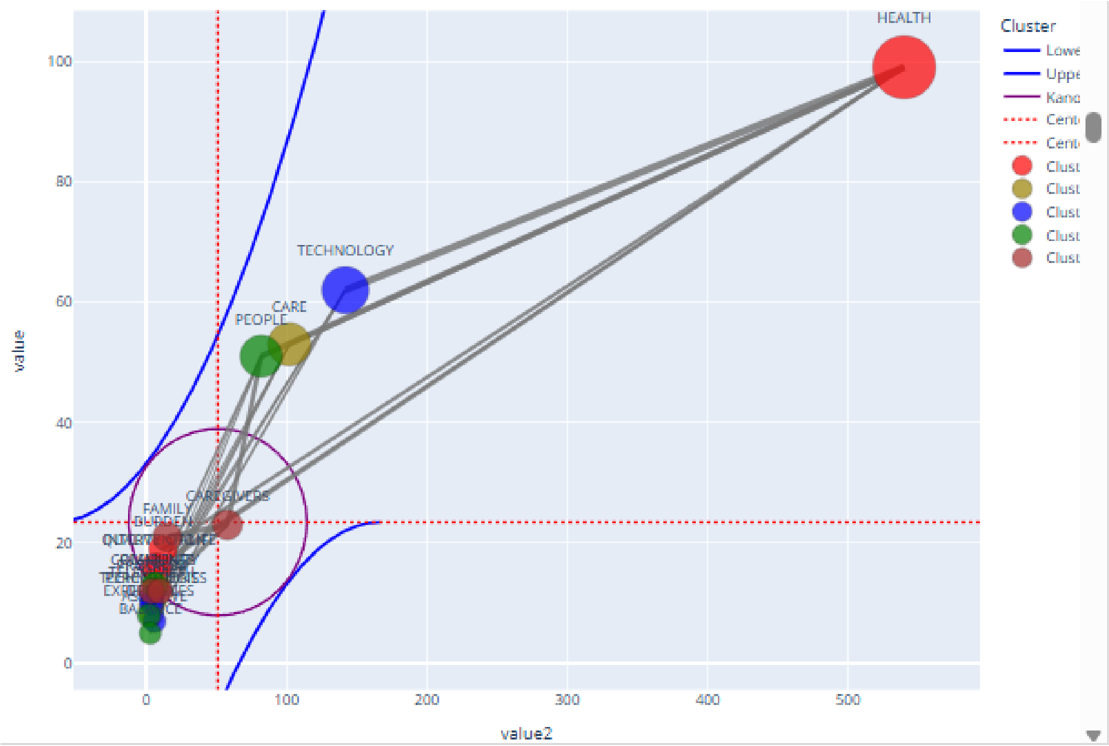

Terms — Network (term labels)

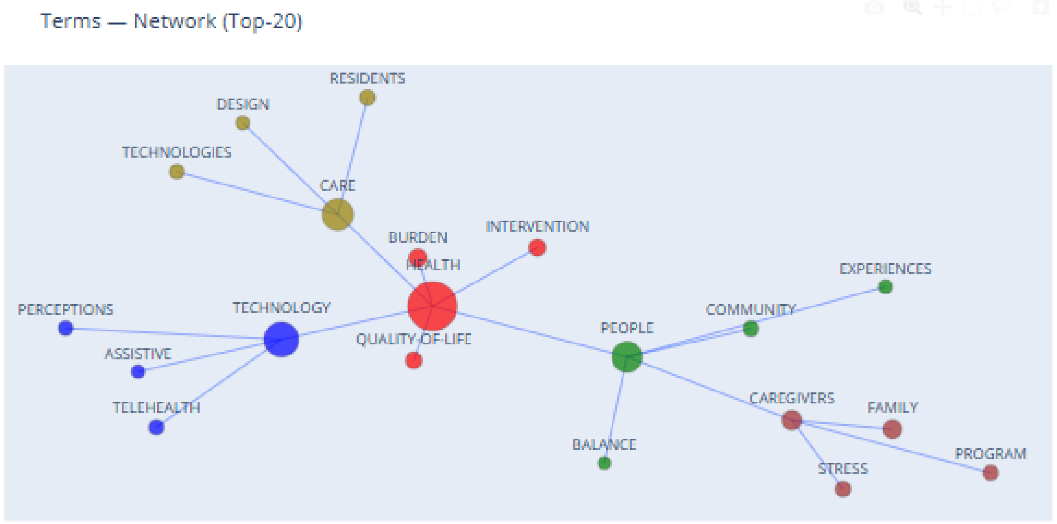

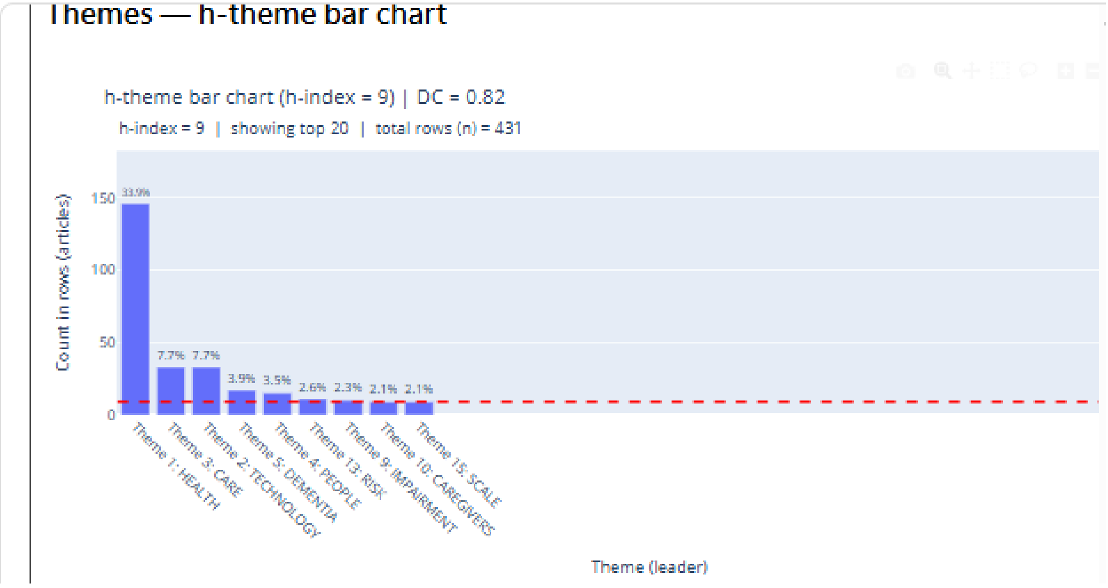
Themes — h-theme bar chart
h-theme bar chart (h-index = 9) | DC = 0.82
h-index = 9 | showing top 20 | total rows (n) = 431
Count in rows (articles)
150
100
50
0
33.9%
7.7%
7.7%
3.9%
3.5%
2.6%
2.3%
2.1%
2.1%
Theme 1: HEALTH
Theme 3: CARE
Theme 2: TECHNOLOGY
Theme 5: DEMENTIA
Theme 4: PEOPLE
Theme 13: RISK
Theme 9: IMPAIRMENT
Theme 10: CAREGIVERS
Theme 15: SCALE
Theme (leader)

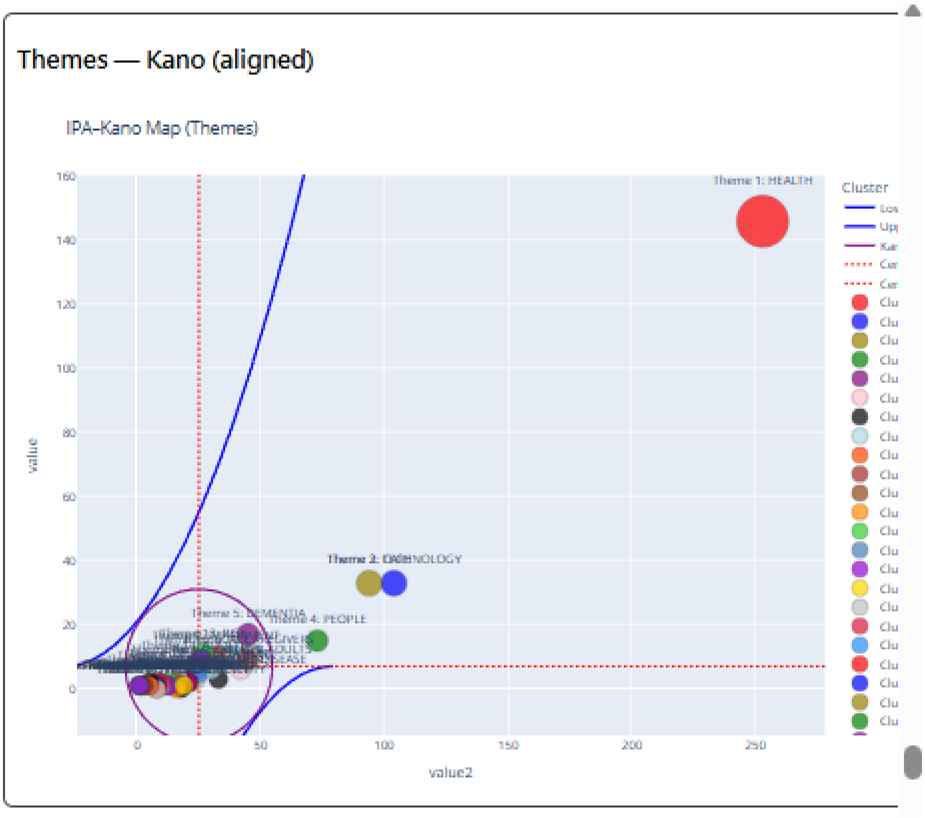
Themes — Kano (aligned)
IPA–Kano Map (Themes)
Theme 1: HEALTH
Theme 5: DEMENTIA
Theme 4: PEOPLE
Cluster
value
value2
160
140
120
100
80
60
40
20
0
0
50
100
150
200
250

Part E — SankeyMATIC (copy/paste & build link)

Open in SankeyMATIC: build link (source: terms_sankeymatic.txt)

Copy SankeyMATIC text

```
HEALTH [20.0000] TECHNOLOGY #000000
HEALTH [16.0000] PEOPLE #000000
CAREGIVERS [15.0000] FAMILY #000000
CARE [13.0000] HEALTH #000000
CAREGIVERS [12.0000] PEOPLE #000000
PEOPLE [12.0000] TECHNOLOGY #000000
CARE [12.0000] PEOPLE #000000
ALZHEIMERS-DISEASE [12.0000] DEMENTIA #000000
ACCEPTANCE [12.0000] TECHNOLOGY #000000
ACCEPTANCE [11.0000] USER #000000
CARE [10.0000] TECHNOLOGY #000000
CAREGIVERS [10.0000] INTERVENTIONS #000000
OLDER-ADULTS [10.0000] TECHNOLOGY #000000
AGING [10.0000] IN-PLACE #000000
HEALTH [9.0000] IMPACT #000000
HEALTH [9.0000] OLDER-ADULTS #000000
HEALTH [9.0000] PREVALENCE #000000
HEALTH [9.0000] QUALITY-OF-LIFE #000000
ACCEPTANCE [9.0000] MODEL #000000
BURDEN [9.0000] HEALTH #000000
FAMILY [9.0000] PEOPLE #000000
DEPRESSION [9.0000] HEALTH #000000
CAREGIVERS [8.0000] STRESS #000000
[illegible]
```

Link to Sankeymatic.com if it is clicked on the icon of build link.

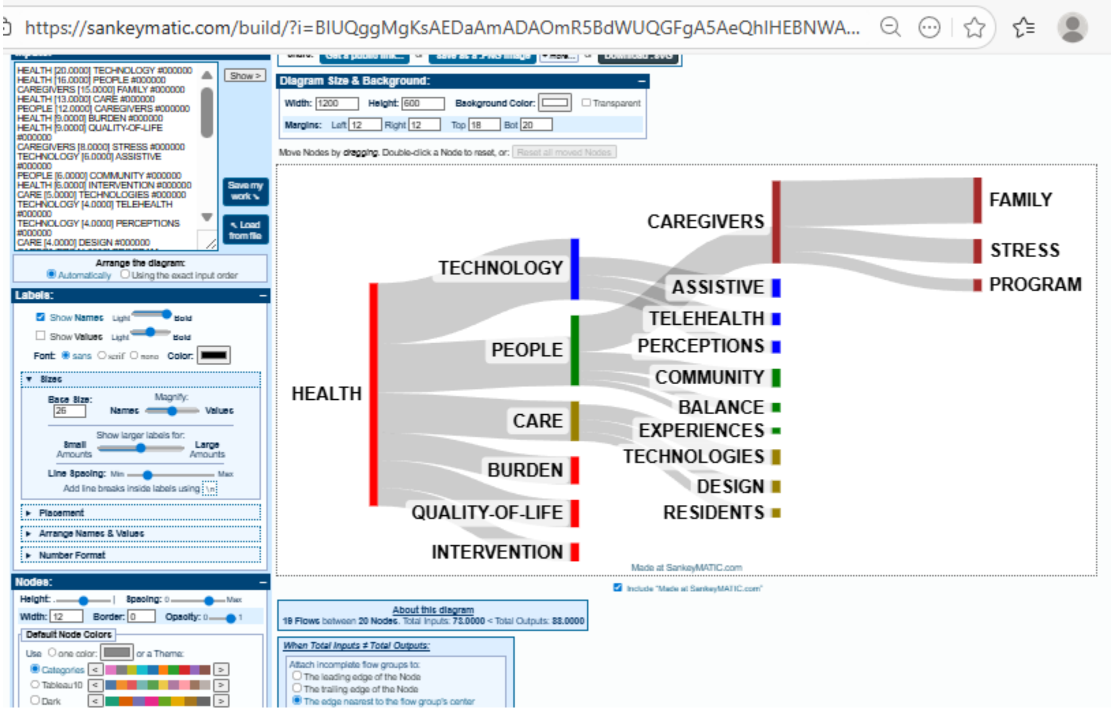

Downloads (table)

File	Description
report.html	report.html (open this)
terms_nodes.csv	terms_nodes.csv
terms_edges.csv	terms_edges.csv (FLCA edges)
terms_edges_raw.csv	terms_edges_raw.csv (raw co-occurrence)
terms_nodes_top20.csv	terms_nodes_top20.csv
terms_edges_top20.csv	terms_edges_top20.csv
top20_relations_raw.csv	top20_relations_raw.csv (Top-20 raw relations)
top20_vertices.csv	top20_vertices.csv (value=sumWCD, value2=edge_count)
top20_relations.csv	top20_relations.csv (raw WCD among top-20 terms)
terms_frequency.csv	terms_frequency.csv (ALL terms)
clusters_summary.csv	clusters_summary.csv (cluster distribution)
theme_assignment.csv	theme_assignment.csv
theme_article.csv	theme_article.csv
theme_nodes.csv	theme_nodes.csv
theme_edges.csv	theme_edges.csv
theme_hindex.csv	theme_hindex.csv (h-theme table)
theme_frequency_all.csv	theme_frequency_all.csv (ALL themes)
inter_rater_sample.csv	inter-rater sample (core themes; fill reviewer labels)
confusion_taaa_vs_reviewer1.csv	confusion (TAAA vs Reviewer 1)
confusion_taaa_vs_reviewer2.csv	confusion (TAAA vs Reviewer 2)
confusion_reviewer1_vs_reviewer2.csv	confusion (Reviewer 1 vs Reviewer 2)

SYSTEM / ROLE

You are a careful semantic classifier. Assign EXACTLY ONE theme from the 9 labels:

HEALTH, CARE, TECHNOLOGY, DEMENTIA, PEOPLE, RISK, IMPAIRMENT, CAREGIVERS, SCALE.

THEME DEFINITIONS (use these scopes; pick best semantic fit)

1) HEALTH: General health status, prevention, wellbeing, comorbidities, symptoms, outcomes (not dementia-specific unless clearly central).

2) CARE: Healthcare delivery/practice, interventions, services, care pathways, quality

of care, care settings, policy/implementation.

3) TECHNOLOGY: Digital health, AI/ML, sensors, apps, telehealth, assistive tech, data systems used to assess/support care/health.

4) DEMENTIA: Dementia/Alzheimer’s/cognitive disorders as the central condition (diagnosis, progression, management, epidemiology).

5) PEOPLE: Population characteristics, demographics, experiences, attitudes, behaviors, engagement, qualitative lived experience (not primarily caregivers).

6) RISK: Risk factors, prediction, screening/risk stratification, odds/associations, incidence determinants.

7) IMPAIRMENT: Functional/cognitive/physical impairment, disability, ADL/IADL limitations, decline measures (when impairment is the focus, not just a symptom).

8) CAREGIVERS: Informal/formal caregivers, caregiver burden/stress, support needs, caregiver interventions, dyads.

9) SCALE: Scale development/validation, psychometrics, reliability/validity, questionnaire/instrument creation, scoring, measurement properties.

TASK

Read the Title + Abstract and select the single most suitable theme.

DECISION RULES

- Use semantic meaning, not keyword counting.

- Base the choice on the PRIMARY contribution (aim + method + main outcome), not background mentions.

- If multiple themes apply, choose the DOMINANT one. Tie-breaker:

  (a) choose the most specific scope; (b) if still tied, choose the earlier item in the list above.

- If none fits well, choose the closest theme but mark weak_fit with low confidence.

- Do not add facts not present in the text.

OUTPUT (JSON only; one line; no extra text)

{

  "theme": "HEALTH|CARE|TECHNOLOGY|DEMENTIA|PEOPLE|RISK|IMPAIRMENT|CAREGIVERS|SCALE",

  "confidence": 0.00-1.00,

  "flag": "ok|multi_theme|weak_fit",

  "why": "<=18 words stating main rationale>",

  "evidence": ["<verbatim phrase from title/abstract>", "<verbatim phrase from

title/abstract>"]

}

EVIDENCE CONSTRAINTS

- Evidence phrases must be copied verbatim from the Title/Abstract.

- Keep both evidence phrases short; total ≤ 25 words.

Summary Report for Performance Analytics of 10-Element Entities Regarding counts, h-index, and AAC for the Top 1 Element Over the Next Two (n=434, h=25)

Entity		RP	FP	n	h
**Country**	AAC=0.54				
U.S	0	135	137	139	17
Canada		63	63	64	14
U.K		33	33	35	9
Australia		26	25	27	7
China		24	21	26	3
Germany		24	24	24	5
Netherlands		20	21	21	7
Hong Kong		14	17	18	4
Japan		11	12	12	4
Singapore		11	10	11	7
**Institure**	AAC=0.56				
Univ Toronto(Canada)		7	8	10	5
Maastricht Univ(Netherlands)		8	7	8	6
McMaster Univ(Canada)		7	8	8	4
Univ Waterloo(Canada)		6	8	8	3
Hong Kong Polytech Univ(Hong Kong)		6	6	7	2
Univ Washington(U.S)		6	5	6	4
Univ Melbourne(Australia)		4	6	6	3
Univ British Columbia(Canada)		5	5	5	3
Imperial Coll London(U.K)		5	4	5	3
Univ Texas Austin(U.S)		5	5	5	3
**Departmen**	AAC=0.45				
Sch Nursing		23		23	5
Nursing		1	22	22	6
Med		11	14	17	6
Neurol		7	8	9	4
Psychiat		3	5	7	2
Coll Nursing		5	6	7	4
Publ Hlth Sci		3	6	7	4
Psychiat & Neuropsychol		7	6	7	6
Social Work			7	7	5
Comp Sci		6	7	7	3
**Author**	AAC=0.67				
Christie Hannah Liane(Netherlands)		6	5	6	5
Shu Sara(U.S)		3	3	3	3
Legare France(Canada)		3		3	2
Sirikul Wachiranun(Thailand)		3		3	
Haase Kristen R.(Canada)		2	1	2	2
Siette Joyce(Australia)		2	1	2	2
LaMonica Haley M.(Australia)		2	2	2	2
Kim Sunyoung(U.S)		2	2	2	1
Huang Bin(U.S)		2		2	1
Guo Yuqi(U.S)			2	2	1
**Keyword**	AAC=0.67				
HEALTH				75	12
PEOPLE				51	13
TECHNOLOGY				48	11
CARE				44	12
OLDER-ADULTS				33	10
DEMENTIA				32	9
ALZHEIMERS-DISEASE				30	7
IMPACT				25	7
ADULTS				20	6
INTERVENTIONS				19	9

Entity			n	h
**Journal**	AAC=			IF
JMIR Aging		Q1|1/48	434	4.8
**Year**	AAC=0.6			h
		2024	130	7
		2025	81	2
		2023	76	9
		2022	68	17
		2021	47	16
		2020	32	13
**Article type**	AAC=0.01			h
Article			376	22
Review			50	15
Correction			5	1
Editorial Material			3	2
**Research area**	AAC=			h
Geriatrics & Gerontology; Medical Informatics			434	25
**Article**	AAC=0.29			citation
Lebrasseur, Audrey(Canada	JMIR Aging		2021	234
Goethals, Luc(France)	JMIR Aging		2020	213
Haase, Kristen R.(Canada)	JMIR Aging		2021	142
Schifeling, Christopher H.(U	JMIR Aging		2020	90
Mao, Alice(U.S)	JMIR Aging		2022	53
Schroeder, Tanja(Australia)	JMIR Aging		2023	51
Tiersen, Federico(U.K)	JMIR Aging		2021	50
LaMonica, Haley M.(Austral	JMIR Aging		2021	49
Bin Zaman, Sojib(Australia)	JMIR Aging		2022	46
Hassan, Alhassan Yosri Ibra	JMIR Aging		2020	41

AAC=γ/(1+γ)

γ=(r1/r2)/(r2/r3) https://rasch.org/rmt/rmt263c.htm

h-index=25

Figure 2. Summary performance report for article metadata categories in JMIR Aging.

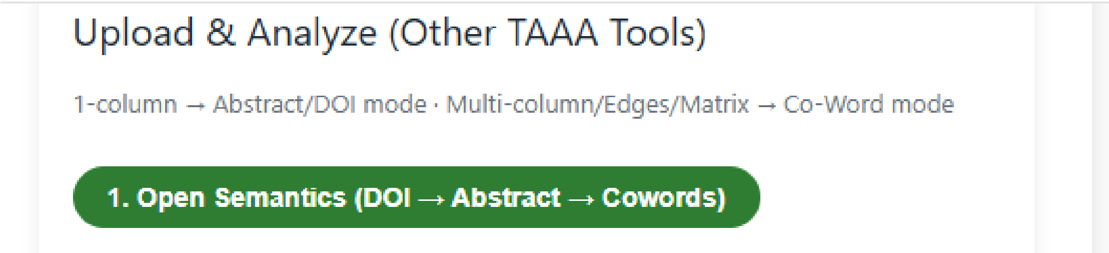

To extract abstract from DOI

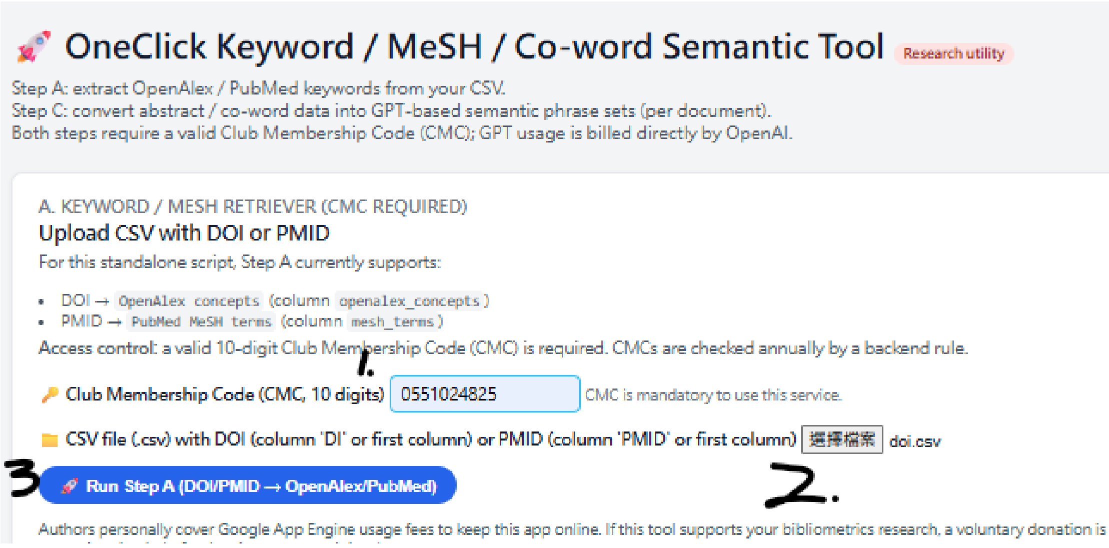

Upload DOI Data to obtain article abstracts

Table 1 Summary of R scrips used in this study with TAAA model

Step	Visualization Method	R script	Figure	Goal
1	Algorithm for Keywords from abstracts	[16]		Classification
2	Bar plot for core themes & TAAA	[17]	2, 3, 4	Assignment
3	Venn diagram	[22]	5	Screening
4	Kano & Sankey Diagrams	[18]	6,7	Leadership

raschonline.com/raschonline/cbp.asp?cbp=NLPkeywordextractioninR

Submit

Tips for R

Copy & Paste the Code in R to Rstudio

Select text

```
  # 載入必要套件
  library(dplyr)
library(tidytext)
library(stringr)
library(udpipe)
library(tidyr)
library(topicmodels)
library(tm)
df <- read.csv("F:/RR/methodsxabstract.csv", stringsAsFactors = FALSE)
df$CombinedText <- paste(df$ArticleTitle, df$Abstract, sep = ". ")
data("stop_words")
df_clean <- df %>%
    mutate(doc_id = row_number()) %>%
    unnest_tokens(word, CombinedText) %>%
    anti_join(stop_words, by = "word") %>%
    filter(!str_detect(word, "\\d")) %>%
    mutate(word = str_to_lower(word)) %>%
    filter(str_length(word) > 3)
tfidf_keywords <- df_clean %>%
    count(doc_id, word, sort = TRUE) %>%
```

Make sure: df <- read.csv("F:/RR/methodsxabstract.csv",

write.csv(df, "F:/RR/methodsxabstract_with_keywords_topics.csv", row.names = FALSE)

in methodsxabstract_with_keywords_topics.csv

	A	B	C	D	E	F	G	H	I
1	ArticleTitle	Abstract	Combined	doc_id	Keywords_TFIDF				
2	Impact of t	Backgroun	Impact of t	1	protective; pandemic; covid; isolation; synthesize; studies; im				
3	Impact of I	Backgroun	Impact of I	2	quarantine; activity; physical; programs; ffpevg; sedentary; co				
4	Older Adul	Backgroun	Older Adul	3	socialization; pandemic; respondents; containment; virus; sam				
5	Disparities	Backgroun	Disparities	4	visits; video; telephone; appointments; visit; telemedicine; pat				
6	Barriers to	Backgroun	Barriers to	5	telemedicine; visits; video; site; barriers; preferred; difficulty				
7	Older Adul	Backgroun	Older Adul	6	intention; digital; technologies; articles; factors; influence; ma				
8	Smart Hom	Backgroun	Smart Hom	7	dementia; occupational; design; smart; centered; opportunities				
9	Understanc	Backgroun	Understanc	8	hits; participatory; mental; meet; appropriateness; technology;				
10	Exploring I	Backgroun	Exploring I	9	chronic; interventions; diseases; enablers; articles; routine; tel				
11	Challenges	Backgroun	Challenges	10	solutions; informal; deployment; challenges; caregivers; recor				
12	Factors Infl	Backgroun	Factors Infl	11	informal; technologies; ehealth; dementia; influencing; impler				
13	Mobile Ap	Backgroun	Mobile Ap	12	apps; mobile; rating; obligation; fees; star; stores				

Separate it with symbol of ;

	A	B	C	D	E	F	G	H
1	A1	A2	A3	A4	A5	A6	A7	A8
2	protective	pandemic	covid	isolation	synthesize	studies	impact	
3	quarantine	activity	physical	programs	ffpevg	sedentary	coronavirus	
4	socializatio	pandemic	respondent	containmer	virus	sampling	columbia	
5	visits	video	telephone	appointmer	visit	telemedicir	patients	
6	telemedicir	visits	video	site	barriers	preferred	difficulty	
7	intention	digital	technologie	articles	factors	influence	mapping	
8	dementia	occupation	design	smart	centered	opportuniti	discussed	
9	hits	participator	mental	meet	appropriate	technology	supportive	
10	chronic	interventio	diseases	enablers	articles	routine	telehealth	
11	solutions	informal	deploymen	challenges	caregivers	recommenc	designers	
12	informal	technologie	ehealth	dementia	influencing	implementa	umbrella	
13	apps	mobile	rating	obligation	fees	star	stores	
14	media	social	singapore	apps	enabled	addiction	bond	compromis

Put it into the top10keywords.csv

Make sure for the file

```
if (1==1){

    # Read the CSV file

    data <- read.csv("F:/RR/top10keywords.csv")
```

} else {

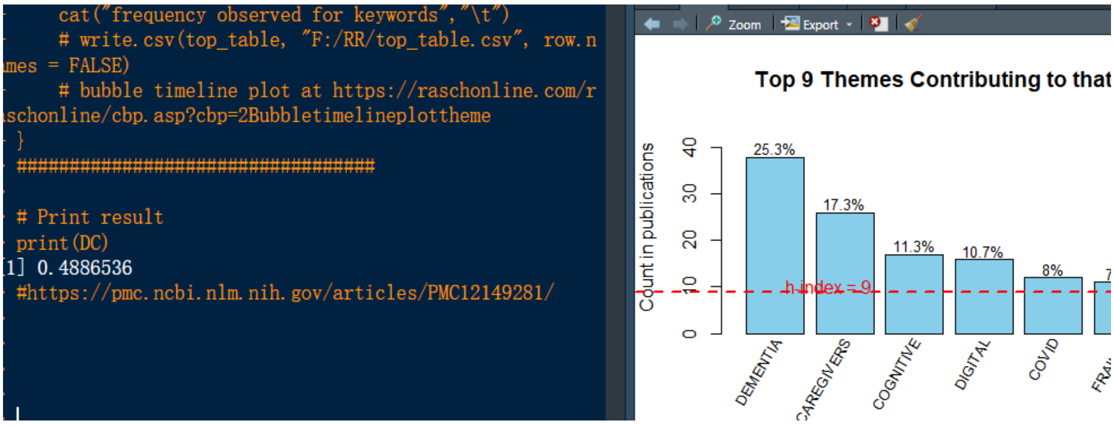

https://raschonline.com/raschonline/cbp.asp?cbp=Venndiagramthen

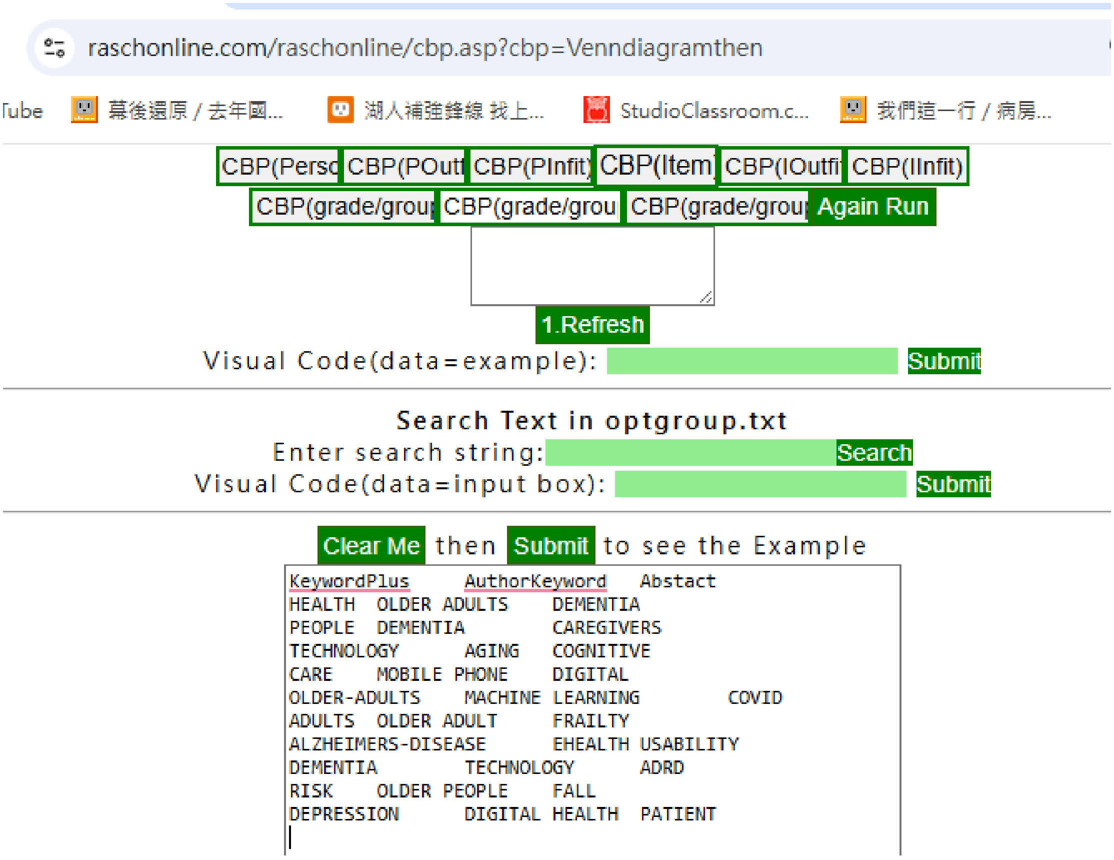

Tips for R

Copy & Paste the Code in R to Rstudio

Select text

```
data<-list(KeywordPlus=c("HEALTH","PEOPLE","TECHNOLOGY","CARE","OLDER-ADULTS","ADULTS","ALZHEIMER
S-DISEASE","DEMENTIA","RISK","DEPRESSION")
,AuthorKeyword=c("OLDER ADULTS","DEMENTIA","AGING","MOBILE PHONE","MACHINE LEARNING","OLDER ADUL
T","EHEALTH","TECHNOLOGY","OLDER PEOPLE","DIGITAL HEALTH")
,Abstact=c("DEMENTIA","CAREGIVERS","COGNITIVE","DIGITAL","COVID","FRAILTY","USABILITY","ADRD","FA
LL","PATIENT")
)

 if (!require(devtools)) install.packages("devtools")
devtools::install_github("yanlinlin82/ggvenn")
library(ggvenn)
ggvenn(data,fill_color = c("#0073C2FF","#EFC000FF","#868686FF"),stroke_size = 0.5, set_name_size
= 4
  )
```

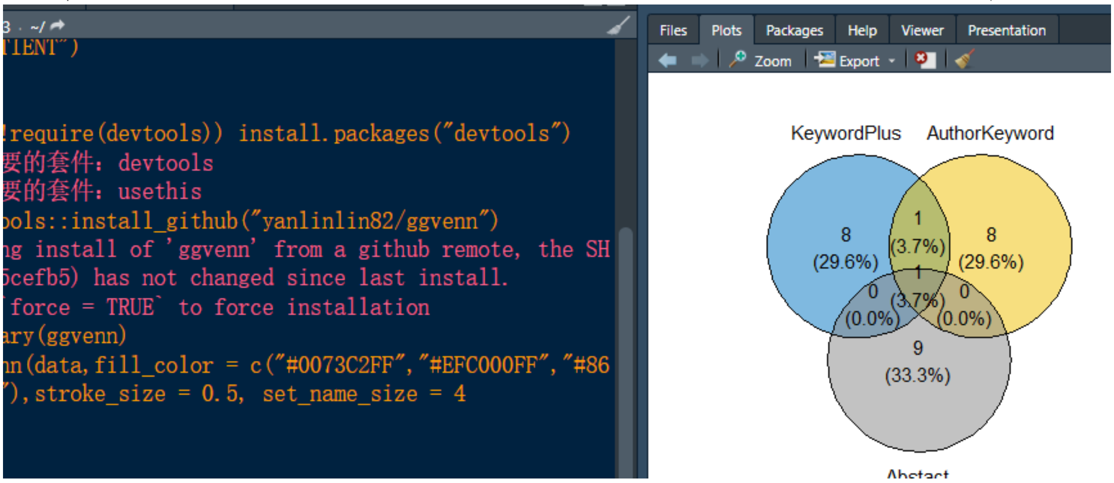

raschonline.com/raschonline/cbpgeo.asp?cbp=FLCAalgorithmexample5

Tips for R

Copy & Paste the Code in R to Rstudio

Select text

```
#

# Load necessary package
library(readr)

# Load the datasets
library(readr)

library(tidyr)
#nodes <- read_csv("F:/RR/nodestemporate.csv", locale = locale(encoding = "UTF-8"))
#nodes <- read_csv("F:/RR/nodestemporate.csv", locale = locale(encoding = "GB2312"))
#####################################
shiefttarget<-1 # 0 <有向性(限二欄) directed arrow   ;無向性1: indirected: larger in target and l
ower caption in source
####################################
FLCAcluster<-1 # if #cluste=1 then 1

ncount<-20
itemlarger20<-ncount
allelement<-FALSE
```

myfile<- "country.csv"

2 columns, 3 columns with WCD, or multipy columns with words

Make sure data in country.csv

	A	B	C	D	E
1	ClusterKey	institute	instituteCoresp		
2	PANDEMI	Ctr Integre	Ctr Integre Univ Sante & Serv Sociau		
3	PHYSICAI	Univ Jean	Univ Jean Monnet(France)		
4	PANDEMI	Univ Britis	Univ British Columbia(Canada)		
5	PATIENTS	Univ Color	Univ Colorado(U.S)		
6	TELEMED	Stanford U	Stanford Univ(U.S)		
7	DIGITAL	Macquarie	Western Sydney Univ(Australia)		
8	DEMENTI	Imperial Co	Imperial Coll London(U.K)		
9	TECHNOL	Univ Sydn	Univ Sydney(Australia)		
10	INTERVEI	Monash Ur	Monash Univ(Australia)		
11	CAREGIV	Italian Natl	Italian Natl Inst Hlth & Sci Ageing(It		
12	DEMENTI	Univ Twen	Univ Twente(Netherlands)		
13	APPS	Univ Ulm(	Univ Ulm(Germany)		
14	SOCIAL	Natl Univ S	Natl Univ Singapore(Singapore)		
15	PHYSICAI	Univ Calif	Univ Calif San Diego(U.S)		

```
addvalue<--1

Importance<-2

myfile<- "country.csv"
##  2 columns, 3 columns with WCD, or
multipy columns with words

parantheses<-1   ## if 1 for add
```

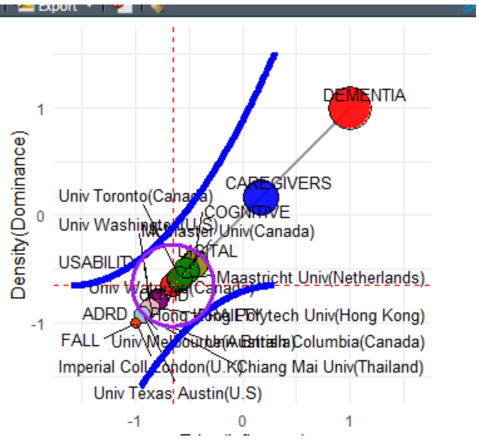

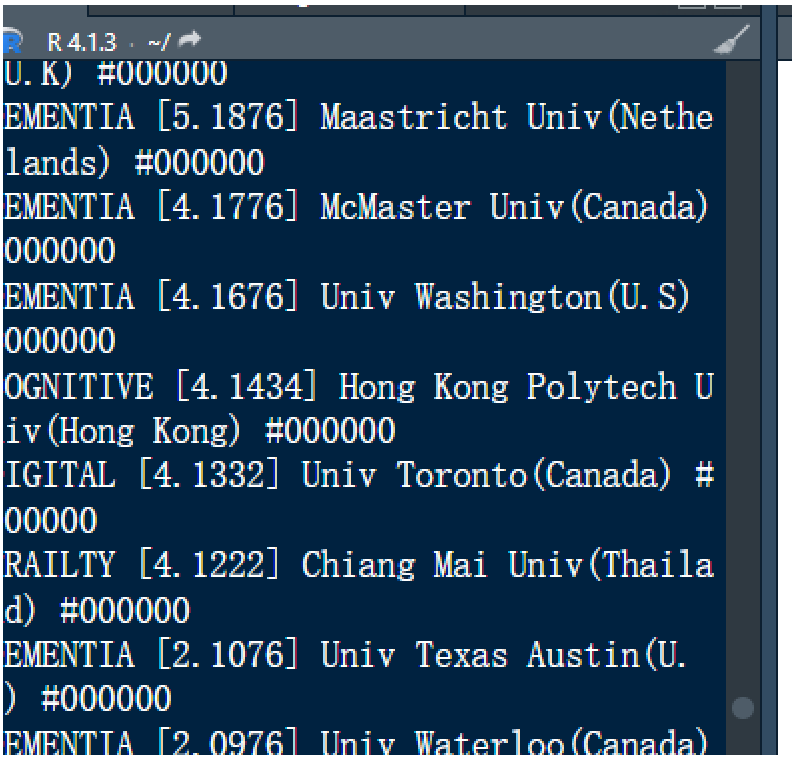

Copy the Sankey code into https://sankeymatic.com/

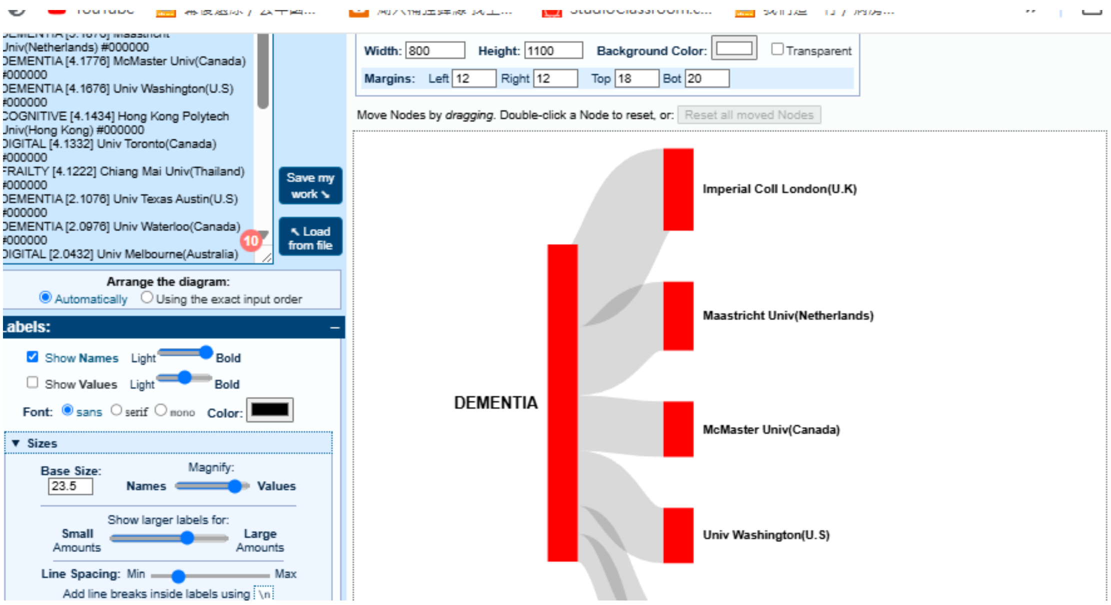

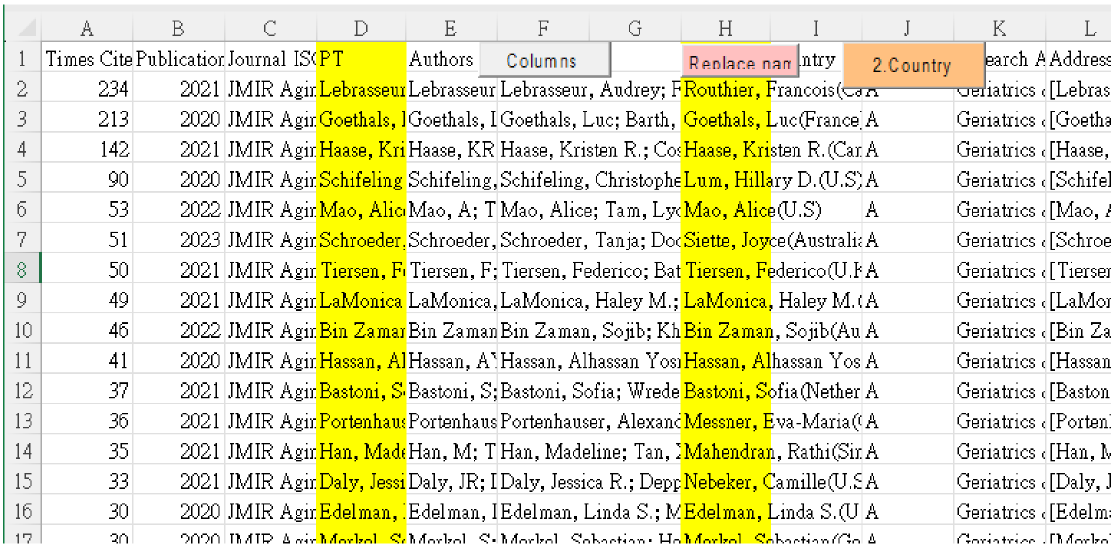

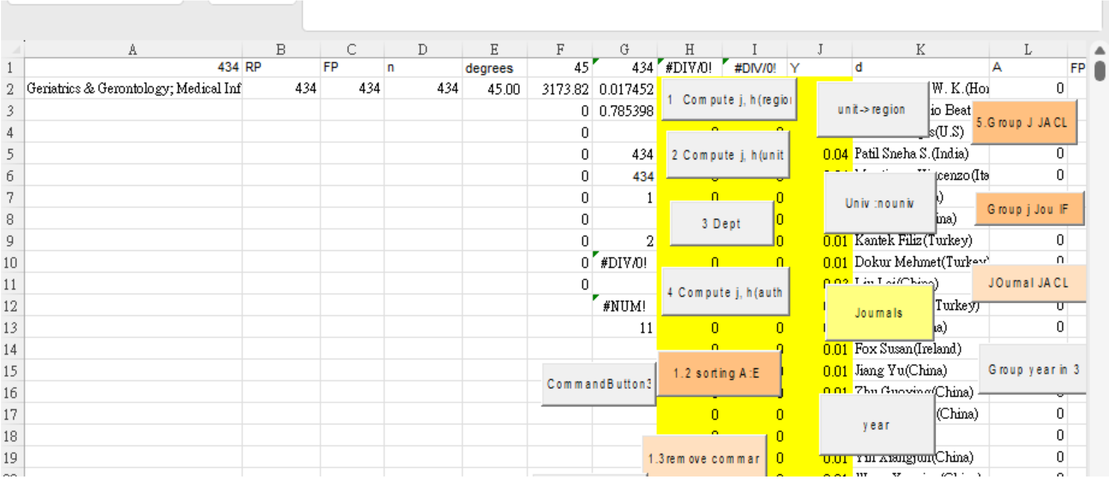

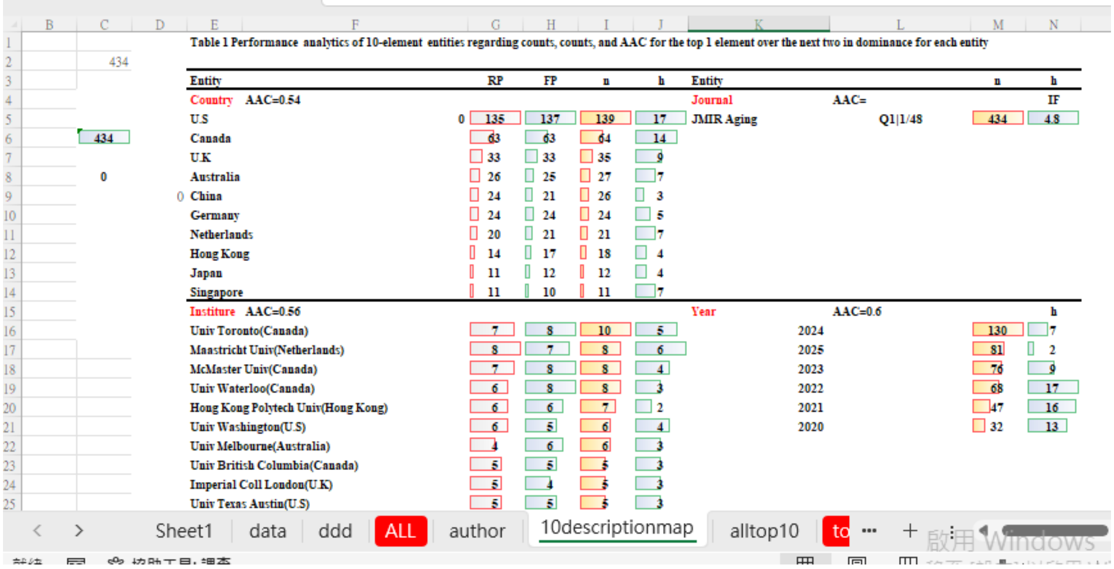

Table 1 Performance analytics of 10-element entities regarding counts, counts, and AAC for the top 1 element over the next two in dominance for each entity

Entity		RP	FP	n	h	Entity			n	h
Country	AAC=0.54					Journal	AAC=			IF
U.S	0	135	137	139	17	JMIR Aging		Q1|1/48	434	4.8
Canada		63	63	64	14					
U.K		33	33	35	9					
Australia		26	25	27	7					
China		24	21	26	3					
Germany		24	24	24	5					
Netherlands		20	21	21	7					
Hong Kong		14	17	18	4					
Japan		11	12	12	4					
Singapore		11	10	11	7					
Institure	AAC=0.56					Year		AAC=0.6		h
Univ Toronto(Canada)		7	8	10	5		2024		130	7
Maastricht Univ(Netherlands)		8	7	8	6		2025		81	2
McMaster Univ(Canada)		7	8	8	4		2023		76	9
Univ Waterloo(Canada)		6	8	8	3		2022		68	17
Hong Kong Polytech Univ(Hong Kong)		6	6	7	2		2021		47	16
Univ Washington(U.S)		6	5	6	4		2020		32	13
Univ Melbourne(Australia)		4	6	6	3					
Univ British Columbia(Canada)		5	5	5	3					
Imperial Coll London(U.K)		5	4	5	3					
Univ Texas Austin(U.S)		5	5	5	3					
